# London Rocket (*Sisymbrium irio* L.) as Healthy Green: Bioactive Compounds and Bioactivity of Plants Grown in Wild and Controlled Environments

**DOI:** 10.3390/molecules30010031

**Published:** 2024-12-25

**Authors:** Tarik Chileh-Chelh, Tatiana Pagan Loeiro da Cunha-Chiamolera, Miguel Urrestarazu, Mohamed Ezzaitouni, Rosalía López-Ruiz, Cinthia Nájera, Miguel Ángel Rincón-Cervera, José Luis Guil-Guerrero

**Affiliations:** 1 Food Technology Division, University of Almería, 04120 Almeria, Spain; chileh@hotmail.es (T.C.-C.); mohamedezzaitouni6@gmail.com (M.E.); mrc883@ual.es (M.Á.R.-C.); 2 Vegetal Production Division, University of Almería, 04120 Almeria, Spain; tatiplcc@ual.es (T.P.L.d.C.-C.); mgavilan@ual.es (M.U.); cnajera4@ual.es (C.N.); 3 Department of Chemistry-Physics, Analytical Chemistry of Contaminants, University of Almería, 04120 Almeria, Spain; rosalialr@ual.es; 4 Institute of Nutrition and Food Technology, University of Chile, Macul, Santiago 7830490, Chile

**Keywords:** London rocket, soilless culture, apigetrin, vitamin C, antioxidant activity, HT-29 cell line

## Abstract

London rocket (*Sisymbrium irio*) is a wild green consumed globally, yet its phytochemical composition remains underexplored. In this study, we analyzed the leaves of wild *S. irio* plants and those grown in controlled environments (GCE) with varying electrical conductivities (EC) and light spectra. Plants were assessed for growth, phenolic content, vitamin C, antioxidant activity, glucosinolates, and antiproliferative effects against HT-29 human colorectal cancer cells. The optimal biomass yield occurred at the EC levels of 3.0–3.5 dS m^−1^ under Valoya^®^ LED light. Wild plants showed higher antioxidant activity (DPPH and ABTS assays) than GCE samples, with values of 8.03–8.67 and 6.49–6.81 mmol TE per 100 g dry weight, respectively. The vitamin C range was 50.7–84.3 and 84.5–186.9 mg 100 g^−1^ fresh weight for GCE and wild samples, respectively. Phenolic content was higher in wild plants than in the GCE ones, with apigetrin as the primary phenolic compound. The MTT assay showed that ethanol extracts from wild plants weakly inhibited HT-29 cell growth, with a GI_50_ of 210–380 µg mL^−1^ after 72 h of cells exposure to plant extracts. Principal Component Analysis suggested that EC and UV exposure increase the antioxidant activity, total phenolics, and glucosinolates in wild plants, offering insights into the bioactive profiles of *S. irio* leaves.

## 1. Introduction

Traditional uses of wild edible plants are being lost throughout generations in most Western cultures [1]. The revalorization of wild vegetables as functional foods could boost their consumption in modern Western diets. A deeper knowledge of wild edible plants, such as London Rocket (*Sisymbrium irio* L.), may improve the biodiversity and rural sustainability of the collection areas.

*S. irio* is an annual species of the Cruciferae (syn. Brassicaceae) family, which includes many globally important crops known for their distinctive flavor and health benefits. *S. irio* itself is widely distributed and found across various regions worldwide. The species of the *Sisymbrium* genus are widely used in traditional medicine, with *S. irio* notably recognized as a medicinal plant in Ayurveda and Unani therapies. It is valued for its antimicrobial, antipyretic, analgesic, and antioxidant properties and is used to treat rheumatism and inflammatory conditions [2,3].

Few reports dealt with leaves from *S. irio*, highlighting its potential as a healthy food due to the amount and diversity of nutrients its leaves contain, such as ascorbic acid and proteins (35%) [4]. *Sisymbrium* species contain flavonoids, alkaloids, anthraquinones, and glycosides [5,6,7,8,9,10]. Al-Jaber (2011) [11] isolated ten flavonoids from the aerial parts of *S. irio*, which were identified as apigenin, luteolin, kaempferol, and several derived glycosides. The lethal dose of the plant extract was higher than 5000 mg kg^−1^ body weight in mice, suggesting that this plant is non-toxic. The above-mentioned author determined the antioxidant activities of several extracts and isolated compounds, highlighting the bioactivity of apigenin-7-*O*-galactoside.

Other phytochemicals in *Sisymbrium* spp. are glucosinolates (GLS), which occur in all *Brassicaceae* species [12]. GLS are secondary metabolites composed of β-thioglucoside-N-hydroxysulphate that is biosynthetically derived from the amino acids occurring in many foods and are widely studied because of their impact on human health [13,14,15]. Medical studies on GLS showed that they improve health and provide protection against serious diseases, such as colorectal cancer, prostate cancer, breast cancer, and myocardial infarction [16]. The mechanism of GLS involves blocking the phase 1 enzyme of the tumor cell cycle while playing an anti-inflammatory role by blocking the release of histamine, which leads to inflammation [15,16].

Literature regarding the bioactivity of *S. irio* leaves is very scarce. ZnO nanoparticles in alcoholic and aqueous extracts were prepared from *S. irio* and were especially rich in phenolics (42.8 mg GAE g^−1^), among other phytochemicals. The particles exhibited high cytotoxic activity against human liver cancer cell lines (HepG2) [17]. The cytotoxic effects of the *n*-hexane, dichloromethane, ethyl acetate, and *n*-butanol leaf extracts were tested against three cancer cell lines: MCF-7 (breast), HCT-116 (colon), and HepG2 (liver). The authors reported that the *n*-hexane fraction showed potent cytotoxic activities against all tested human cancer cell lines, while the dichloromethane fraction was particularly potent against HCT-116 cells (IC_50_ 5.42 μg mL^−1^) [18].

In most plants, whether vegetables or wild species, nutrient uptake and phytochemical composition often respond to biotic and abiotic stress factors, including salinity levels and light radiation [19,20]. Salinity is directly linked to one of the major abiotic stresses affecting crops [21,22]. Although there is abundant information on the effects of salinity of nutrient solutions on the nutrient composition and phytochemical accumulation in many crops [23,24,25], there is very little information on the influence of salinity on the phytochemical properties of wild edible plants, such as *S. irio*. Light conditions are also highly influential on the morpho-physiology of plants and on the biosynthesis and accumulation of phytochemicals, especially in controlled growth environments [19]. Furthermore, the light intensity of LEDs can be fine-tuned to increase the growth and phytochemical composition of plants [26,27]; thus, LEDs are being implemented in different horticultural areas, for instance, in studies regarding photoperiod lighting for greenhouses [28,29]. Overall, LED lighting systems have significant advantages over traditional lighting due to their spectral composition, durability, wavelength specificity, low radiant heat, and energy efficiency [20]. In this regard, the influence of different LED lights on wild plants has been tested, as in Ice Plant (*Mesembryanthemum crystallinum* L.) [30] and Slender Amaranth (*Amaranthus viridis* L.) [31] plants. It was found that NS1, NS12, and AP67 lamps induced a positive response concerning white light for both vegetative growth and phytochemical composition [30,31].

Although *S. irio* collected from the wild is consumed worldwide, there is a lack of knowledge on its adaptability to different cultivation conditions. Research on this subject aims to determine whether it could be supplied in quantities that could satisfy the needs of consumers in the event it becomes a vegetable for regular consumption. Consequently, this work is focused on disentangling the phytochemical composition, antioxidant activity, and in vitro antitumor activity against colorectal cancer cells of *S. irio* leaves derived from wild plants and those grown in a controlled environment (GCE) and seeks to unravel their health benefits.

## 2. Results

### 2.1. Effect of Salinity and Light on Growth Parameters in Cultivated S. irio

The volume fraction of fertigation to drainage was maintained between 0.2 and 0.3 to ensure that the matric potential of water in the substrate remained constant [32]. Electrical conductivity (EC) values (expressed in dS m^−1^) one and two units above the fertigation value are considered adequate to maintain an osmotic pressure in the rhizosphere [33]. In the EC experiment, the EC of drainage was partially above the levels that could be considered optimal, as noted in Supplementary Figure S1a. The drainage ECs throughout the crop remained constant with different mean values depending on each EC treatment [33]. The lowest (2.0 dS m^−1^) and highest (4.0 dS m^−1^) EC treatments showed the lowest drainage EC values, while the intermediate EC treatment (3.0 dS m^−1^) had the highest values (Supplementary Figure S1b). These results disagree with those reported in Slender Amaranth by Cunha-Chiamolera et al. (2024) [30], who recorded a continuous increase in the drainage EC as the salt concentration in the fertigation solution increased. In this work, the drainage pH followed a trend similar to EC. It varied significantly among treatments with a constant fertigation value of 5.8 (Supplementary Figure S1c). The pH value of treatment C3 (nutrient solution at EC of 3.0 dS m^−1^) was higher than that of other treatments, with an average pH of 7.7 (Supplementary Figure S1d). pH levels up to one or two units above the fertigation pH value are expected under normal vegetative growth conditions, indicating a high metabolic activity in the absorption of nutrients [33].

Considering the lighting trial, significant differences were also found in the fertigation monitoring parameters. Except for treatment L1 (Control, T8 Roblan^®^), which showed an increase in EC in the last week, the EC values of the remaining drainages did not show significant differences (Supplementary Figure S2a). The mean value was 2.9 dS m^−1^ (Supplementary Figure S2b). Except for the drainage pH value in L4 (L18 NS12 Valoya^®^), which was constant during the experiment, there was a general increase in drainage pH values for all treatments (Supplementary Figure S2c). The highest pH values of drainage were observed in treatments L3 and L4, while L1 and L2 had the lowest values (Supplementary Figure S2d). In this sense, an average pH threshold one unit above the fertigation value (5.8) is expected under normal growing conditions [33,34].

**Figure 1:**
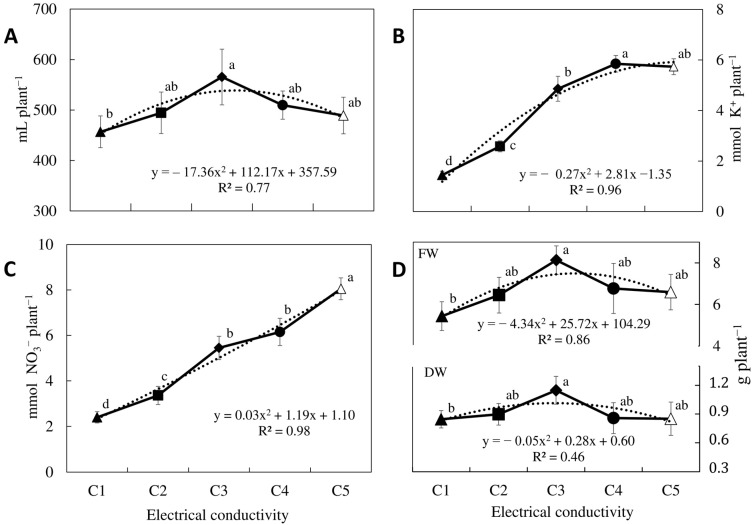
Water (**A**), potassium (**B**), and nitrate uptake (**C**) and fresh (FW) and dry weight (DW) (**D**) of *S. irio* grown under a soilless culture system versus different ECs of the nutrient solutions (C1, C2, C3, C4, and C5, corresponding to 2.0, 2.5, 3.0, 3.5, and 4.0 dS m^−1^, respectively). The dotted lines indicate the trend in the data. Different letters within the same series indicate significant differences among ECs (*p* < 0.05).

There was a statistically significant difference among values of fertigation uptake in the EC experiment (Figure 1A), and the maximum absorption value was noted at an EC of 3.0 dS m^−1^. The EC values one unit less or one unit more caused a different absorption loss, while reducing the salt concentration of the fertigation caused a decrease of 19% in uptake; increasing this same unit led to a 13% decrease. This pattern of fertigation volume uptake agrees with the classical growth model of Sonneveld and Voogt (2009) [35] and with that noted in Ice Plant [31]. However, a continuous linear increase in the uptake of potassium and nitrate was found when the EC of the solution and the content of these ions increased in the nutrient solution (Figure 1B,C), which agrees with that reported by Gallegos-Cedillo et al. (2016) [36], who also found a steady increase in nitrate and potassium uptake as their concentration in the nutrient solution increased, regardless of the increase in EC.

**Figure 2:**
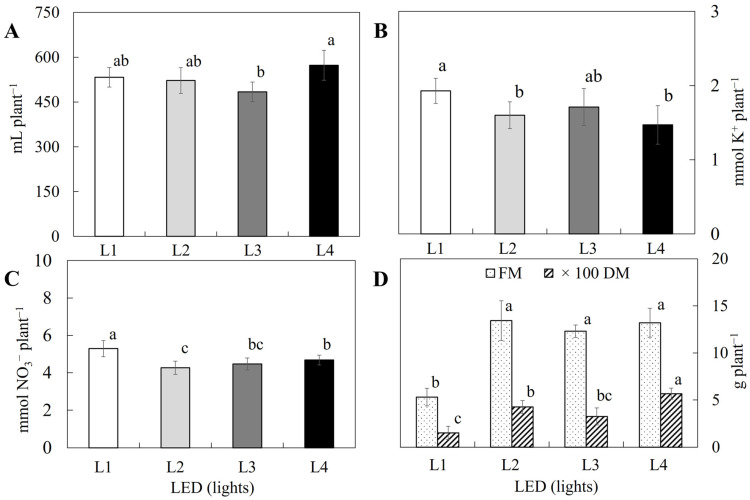
Water (**A**), potassium (**B**), and nitrate uptake (**C**) and fresh (FW) and dry weight (DW) (**D**) of *S. irio* grown under a soilless culture system versus spectrum of illumination. L1: L18 T8 Roblan^®^; L2: L18 AP67 Valoya^®^; L3: L18 NS1 Valoya^®^; and L4: L18 NS12 Valoya^®^. Different letters within the same series indicate significant differences among LEDs.

The growth response (fresh weight and dry weight) behavior showed agreement with the classic production model of Mass and Hoffman (1977) [21] and Sonneveld and Voogt (2009) [35], where the threshold of decrease in production was 3.5 dS m^−1^. The yield decreased per unit of the increase in salinity beyond the threshold of 18.9%. The optimum yield (fresh and dry) of *S. irio* plants was recorded from 3.0 to 3.5 dS m^−1^ (Figure 1D). Water, potassium, and nitrate absorption differed significantly depending on the applied light spectra (Figure 2A–C). Lamps with spectra specifically designed for agronomy did not have a clear effect on absorption in fertigation, which disagrees with that reported by Cunha-Chiamolera et al. (2024) [30] and Rincón-Cervera et al. (2024) [31] for Slender Amaranth and Ice Plant using the same spectra.

In contrast, the spectra designed for use in horticulture significantly influenced the growth of *S. irio* plants (Figure 2D), which had a growth average of 58.6%, and L4 induced the highest growth, in agreement with the results of Rincón-Cervera et al. (2024) [31].

### 2.2. Moisture

Table 1 presents the moisture content of *S. irio* samples, either wild or cultivated plants, the latter exposed to various salinity and light conditions. Wild samples (WD, WG, WM, and WT) had a moisture content exceeding 82%, with WG standing out at 86.1%. In contrast, the cultivated plants under saline conditions (C1–C5) showed a significantly lower moisture content, reaching a minimum of 80.1% in C5. On the other hand, the light treatments (L1–L4) induced moisture values below 82%, especially L3 (80.3%).

### 2.3. Vitamin C Content

Values for vitamin C are detailed in Table 1. There was a significant variation in vitamin C concentrations in *S. irio* between wild and cultivated plants. Wild samples showed values from 50.7 (WG) to 84.3 mg 100 g^−1^ fw (WT). In contrast, the cultivated samples under various salinity levels showed the highest values, in the 84.5 (L1)-193.2 (C3) mg 100 g^−1^ fw range. On the other hand, light treatments induced intermediate vitamin C concentrations, with L2 standing out (113.9 mg 100^−1^ g fw).

### 2.4. Antioxidant Activity

The antioxidant activity of *S. irio* is detailed in Table 1. Wild samples showed DPPH values from 8.03 (WD) to 8.67 (WG) mmol TE 100 g^−1^ dry weight (dw), and the ABTS assay values were between 6.49 (WT) and 6.81 (WG) mmol TE 100 g^−1^ dw. In contrast, the samples from saline treatments showed lower and similar DPPH figures, in a range of 6.14 (C1)–7.25 (C4) mmol TE 100 g^−1^ dw, and lower ABTS values, from 4.14 (C1) to 6.14 (C5) mmol TE 100^−1^ dw. In the lighting experiment, treatment L1 displayed the highest DPPH (7.44 mmol TE 100^−1^ g dw) and L4 the highest ABTS (6.28 mmol TE 100 g^−1^ dw) values. Pure compounds tested with the DPPH method showed values between 17.6 (α-tocopherol) and 23.4 mmol TE 100 g^−1^ (vitamin C), while ABTS assay results ranged from 8.8 (α-tocopherol) to 10.5 mmol TE 100 g^−1^ (vitamin C).

### 2.5. Total Phenols and Flavonoids

Table 1 presents the total phenolic content (TPC) and total flavonoid content (TFC) in the various *S. irio* samples. Wild samples, particularly the WT one, showed the highest TPC (575.4 mg GAE 100 g^−1^ fw) and TFC (336.8 mg QE 100 g^−1^ fw) values. In GCE samples, a notable variability in the TPC and TFC levels was noted alongside the various treatments. Among saline treatments, C3 showed a TPC of 455.2 mg GAE 100 g^−1^ fw and a TFC of 205.4 mg QE 100 g^−1^ fw. In contrast, samples subjected to light treatments had lower TPC and TFC, with L4 having the highest figures—TPC of 240.7 mg GAE 100 g^−1^ fw and TFC of 156.7 mg QE 100 g^−1^ fw.

### 2.6. Phenolic Compound Profiles

The phenolic compound profiles quantified through the HPLC–DAD system are detailed in Table 2, while the results obtained by the LC–MS system are detailed in Supplementary Table S1. The phenolic compounds quantified by the HPLC–DAD system were hydroxycinnamic acids (*trans*-ferulic and *trans-p*-coumaric acids), benzoic acid derivatives (protocatechuic acid), and flavonoids. All phenolic acids were detected in low concentrations. As for flavonoids, most of them were detected in low amounts. The predominant compound in all cases was apigetrin (apigenin-7-*O*-glucoside), which ranged from 3.1 (L4) to 86.8 mg 100 g^−1^ (WT), followed by apigenin, which was between 1.9 (L4) and 15.6 mg 100 g^−1^ (WG). Quantified through the sum of peak areas, the TFC ranged from 6.0 (L4) to 98.4 (WT) mg 100 g^−1^, and the TPC ranged from 6.4 (L4) to 110.7 (WT) mg 100 g^−1^.

The identification of compounds in *S. irio* samples through HPLC–DAD was further validated by LC–MS analysis. In addition to the compounds detected by HPLC–DAD, the LC–MS system enabled the detection of additional compounds for which no standards were available (Supplementary Table S1). These compounds were tentatively identified in the HPLC–DAD chromatogram based on their retention time and absorbance (nm) and were quantified using structurally similar compounds, as detailed in Table 2. The phenolic compounds identified included gallocatechin (−), isoquercetin, isorhoifolin, isorhamnetin-3-*O*-glucoside, and nicotiflorin (Table 2). Furthermore, other phenolic compounds were detected based on their *m*/*z* ions and fragment patterns, although they were present in trace amounts and did not show clear peaks in the HPLC–DAD chromatogram (Supplementary Table S1). Considering that all prominent peaks in the HPLC–DAD chromatogram were successfully assigned, the unquantified compounds occurred in minor amounts, and these were 4-hydroxybenzoic acid, vanillic acid, procyanidin B1, chlorogenic acid, caffeic acid, epicatechin (−), delphinidine, narirutin, pelargonidine, astragalin, sinapic acid, isovitexin, and narcissin.

### 2.7. Glucosinolates

**Figure 3:**
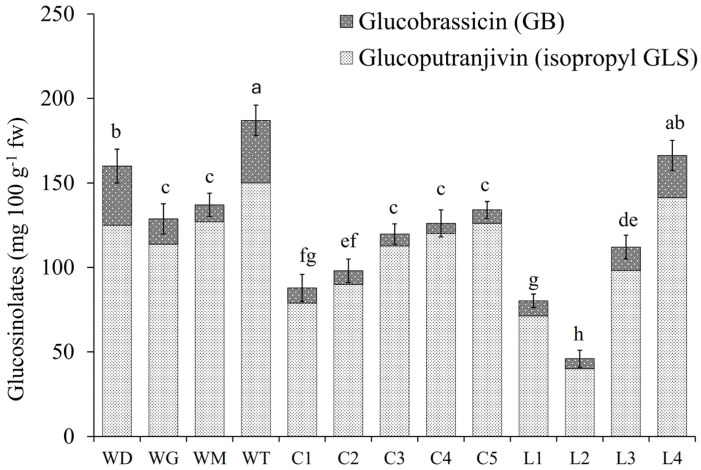
GLS profiles of *S. irio* shoots. Wild plants: WD, WG, WM, and WT. Samples from the EC trial: C1–C5. Samples from the lighting experiment: L1–L4. In a bar, means followed by different lower-case letters are significantly different at *p* < 0.05.

Two types of GLS, glucobrassicin (GB) and glucoputranjivin, were identified in *S. irio* leaves (Supplementary Table S2), and the GLS profiles are shown in Figure 3. Total GSL content ranged from 46 (L2) to 187 (WT) mg 100 g^−1^ fw. In wild plants, the range was between 129 (WG) and 187 (WT) mg 100 g^−1^ fw; in the CE experiment, GSL ranged from 88 (C1) to 134 mg 100 g^−1^ fw (C5); and in the lighting experiment, the GSL content was from 46 (L2) to 166 (L4) mg 100 g^−1^ fw. The main GSL in all samples was glucoputranjivin, which ranged from 40 (L2) to 150 (WT) mg 100 g mg 100 g^−1^ fw, while glucobrassicin ranged from 6 (C4 and L2) to 35 (WD) mg 100 g^−1^ fw.

### 2.8. Principal Component Analysis

**Figure 4:**
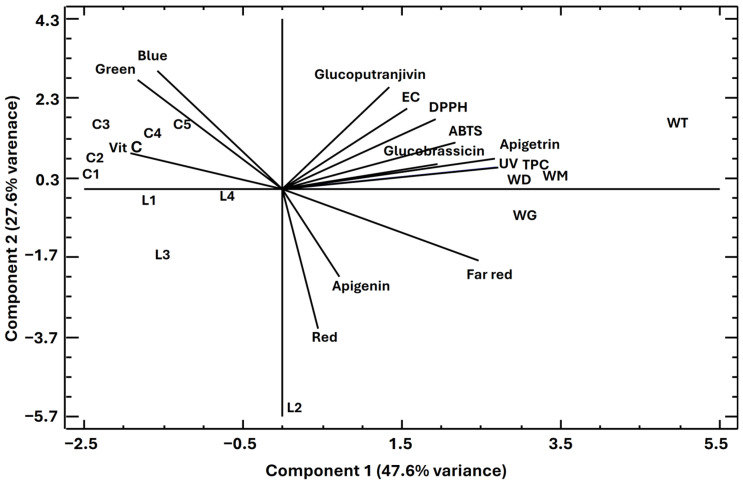
Biplot for the two first component weights. Wild plants: WD, WG, WM, and WT. Samples from the EC trial: C1-C5. Samples from the lighting experiment: L1–L4.

To investigate the possible relationship among the analyzed parameters and sample characteristics, Principal Component Analysis (PCA) was performed. PCA was carried out using data for all variables shown in the plot. In this analysis, 14 components were extracted. Together, they explain 100.0% of the variability in the original data. In this analysis, the two first components explained 47.6 and 27.6% of the total variance, respectively, representing 75.2% of the total variance. Among the different graphics offered by the PCA tools, the biplot allowed a clearer interpretation. The horizontal axis represents PC1, and the vertical axis represents PC2. Upon observing the components through geometrical representation, it is possible to notice that the samples can be grouped according to their values for the measured variables. Figure 4 shows the projection of the PCA biplots on the plane formed by the first two principal components: PC1 and PC2.

### 2.9. Antiproliferative Activity of S. irio Extracts

**Figure 5:**
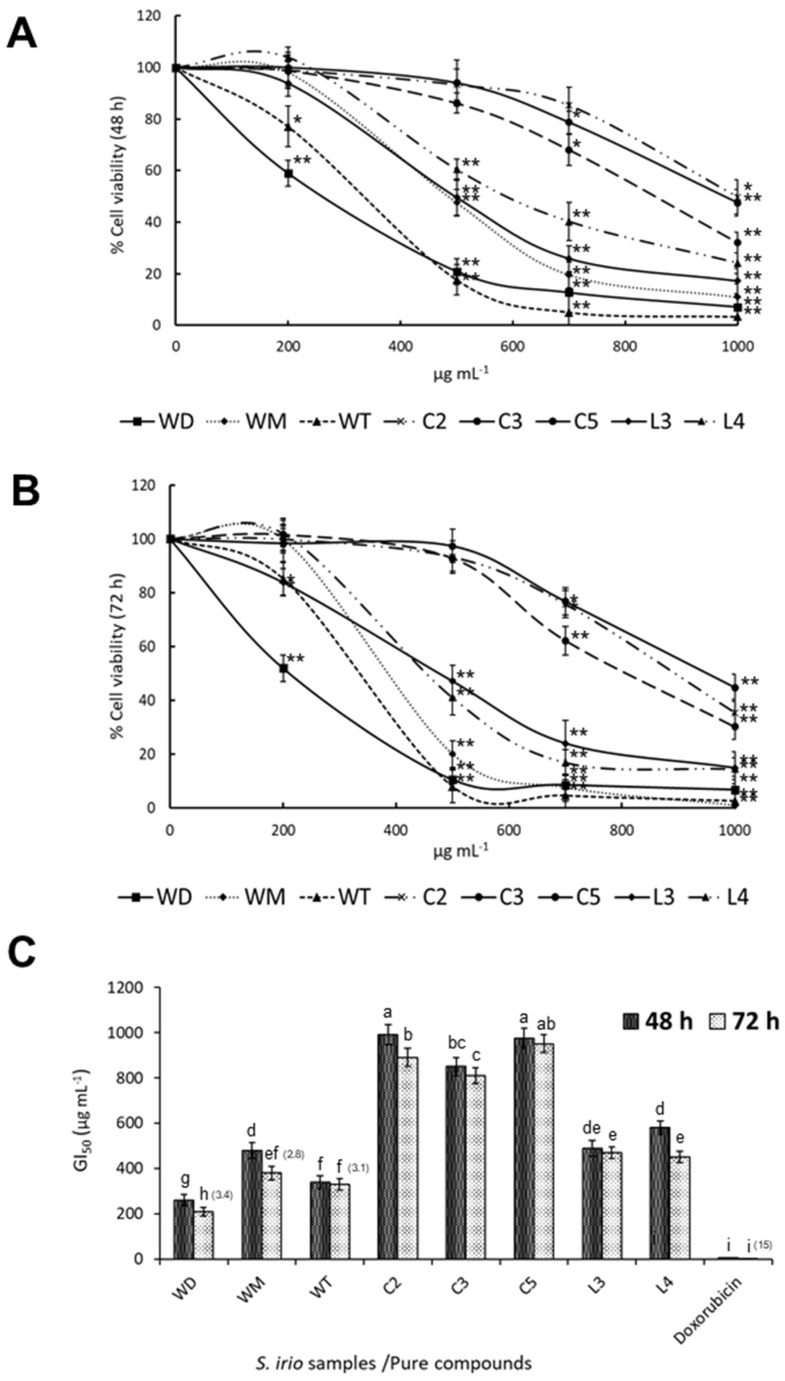
MTT assay. Dose–response curves of HT-29 cell viability after treatment with different concentrations of phenolics- and glucosinolates-containing dry hydroalcoholic leaf extracts from *S. irio* collected in the wild (WD, WM, and WT) and GCE plants subjected to different EC treatments (C2, C3, and C4) and light treatments (L3 and L4) after 48 h (**A**) and 72 h (**B**) of cell exposure to plant extracts (µg of dry extract per mL of culture medium); (**C**) GI_50_ values of WD, WM, WT, C2, C3, C4, L3, and L4 plant extracts and doxorubicin. Data represent the mean of three complete independent experiments ± SD (error bars), with statistical significance equal to * *p* < 0.05 and ** *p* < 0.01. In a bar, means followed by different lower-case letters are significantly different at *p* < 0.05. The Selectivity Index (SI) for cells exposed to plant extracts is shown in parentheses in (**C**).

To assess the antiproliferative activity of the ethanol extracts from *S. irio* leaves on HT-29 human colorectal cancer cells, the MTT assay was conducted (Figure 5). Extracts from wild and cultured samples obtained under different light and salinity conditions were checked. The effects of extracts on cell viability after 48 and 72 h of treatment are shown in Figure 5A,B. The extracts from WD, WM, WT, C2, C3, C5, L3, and L4 at 1000 µg mL^−1^ fw and after 72 h of exposure to cells resulted in reductions of 93.1, 99.0, 97.3, 64.5, 69.7, 55.2, 65.0, and 85.4%, respectively, in cancer cell viability compared to the control group (without extract addition). Figure 5C depicts the GI_50_ (the dose of extracts that inhibited cell growth by 50%) of the more active samples. The GI_50_ of doxorubicin is also displayed. After 72 h of incubation, WD, WM, WT, C2, C3, C5, L3, and L4 showed GI_50_ values of 210, 380, 330, 890, 810, 950, 470, and 450 µg mL^−1^, respectively. The positive control doxorubicin had a GI_50_ of 3 µg mL^−1^. The SI of HT-29 versus normal CCD-18 cells was assessed after 72 h of cell exposure to extracts and pure compounds having GI_50_ < 400 µg mL^−1^, and this index was 2.8 (WM), 3.1 (WT), and 3.4 (WD) for *S. irio* extracts, and for doxorubicin, it was 15.

## 3. Discussion

### 3.1. Effect of Salinity and Light on Growth Parameters in S. irio

Usually, the fraction of volume, EC, and pH of the drainage are parameters used to control nutrient solution management and fertigation [23,37]. In the EC experiments performed in this work, the drainage EC values were above the acceptable limits, i.e., one or two units of dS m^−1^ above the value applied in the nutrient solution. There was a clear tendency for nitrate and potassium uptake to increase in parallel with a rise in the EC of the nutrient solution, and this trend indicates that the higher the EC, the greater the metabolic activity of the root uptake of potassium and nitrate [22,36,38]. On the other hand, in the lighting experiment, the EC values of drainage were also within the acceptable limits [33]. The effect of pH on the growth and nutrient uptake of plants was established long ago and is still being reported (e.g., [20,39,40,41]). Maintaining the correct pH in the rhizosphere implies controlling the pH of fertigation according to the drainage pH, where one or one and a half pH units above that applied in fertigation is suitable for the vegetative growth [33,35]. The results obtained for the crop were within the acceptable limits.

It is well known that there is a very high correlation between the fertigation absorption (especially in the volume of water absorbed) and the final production of the crop [31,34]. Water uptake has been described as a parameter that is highly correlated with growth and yield, while nitrate and potassium are used as indicators of adequate mineral uptake [20]. The results of the EC experiment corroborated that the treatments with better water absorption were the ones that showed higher production in both fresh and dry weight. A clear quadratic model between the EC values in the root environment and the yield of *S. irio* was obtained. The optimum values of 3.0–3.5 dS m^−1^ for fertigation and the yield of *S. irio* indicate this vegetable is moderately tolerant to salinity following the criterium of the classical model of Mass and Hoffman (1977) [21], adjusted according to Sonneveld [42], in which a loss rate relative yield under non-saline conditions (33%, from 0 to 2.5 dS m^−1^) was higher than the yield reduction slope (19%, in % per unit EC in dS m^−1^) caused by excess salinity (>3.5 dS m^−1^). Thus, *S. irio* could be classified as moderately sensitive to salinity or a halophyte crop, like squash, beet, and zucchini. On the other hand, higher nutrient uptake of nitrate and potassium was related to an increased availability of these nutrients in the fertigation, leading to greater uptake by the plant [36].

The lighting experiment was designed as a large number of research papers suggest that lamps with spectrums more closely matched to the requirements of horticultural plants show 20 to 30% better results than LED lighting used for human room lighting purposes [43,44]. Light spectral composition and intensity are important factors related to crop yield, and tailored light conditions can enhance plant growth and productivity [27,29]. However, the results of this work indicate that these conclusions depend on the specific cultivar or species. In this sense, in *S. irio*, L1 and L4 lamps gave the best results for water, nitrogen, and potassium uptake. Previously, Cunha-Chiamolera et al. (2024) [30] and Rincón-Cervera et al. (2024) [31], working with Slender Amaranth and Ice Plant, obtained better performance using L2, L3, and L4 lamps than with L1. Usually, treatments with specific lights for agriculture (L2, L3, and L4) show approximately higher growth (60%) than that obtained for control (L1). In *S. irio* plants, while the illumination spectrum was consistent with previous trials using the same illuminants [44], it showed different results for metabolic indicators (water and nutrient absorption) than expected [20]. Such divergent results for different plants suggest that the effects of the light spectrum on plant performance are species-dependent; hence, the best spectrum for each species should be specifically evaluated.

### 3.2. Vitamin C Content

A significant variability in vitamin C levels among the various *S. irio* samples was found. Wild plants exhibit lower vitamin C concentrations (50.7–84.3 mg 100 g^−1^ fw) than GCE plants (84.5–193.2 mg 100 g^−1^ fw). Previously, it has been reported that vitamin C was generally higher or similar in cultivated plants than in their wild counterparts, such as in *Mesembryanthemum nodiflorum*, *Suaeda maritima*, *Sarcocornia fruticosa,* and *A. viridis* [30,45]. This fact could be explained by considering the absence of metabolic stress induced by a nutrient or water deficit, which can occur under specific growing conditions in the wild, and this would give rise to plants with very different concentrations of vitamin C. In this work, in the EC experiment, plants grown with EC higher than 2.5 dS m^−1^ showed higher concentrations of vitamin C, reaching a maximum at an EC of 3.0 dS m^−1^ (193 mg 100 g^−1^ of vitamin C). In contrast, it was unfeasible to associate the spectrum of any lamp with vitamin C concentration. In wild-collected *S. irio*, 61–68 mg 100 g^−1^ of vitamin C have been reported [4,46]. In these works, vitamin C was determined using spectrophotometric procedures, and values agree with the vitamin C levels found in this work, in which the determination was carried out using the HPLC–DAD methodology.

### 3.3. Antioxidant Activity

According to Floegel et al. (2011) [47], the noted differences in the antioxidant activity determined by various methods may be mainly due to the polarity of the antioxidants present in the tissue matrix and the basis of the chemical action of each of the methods. The ABTS assay is based on the generation of a free radical that allows quantifying this biological activity in both hydrophilic and lipophilic antioxidant systems, so it has been reported that this method in most plant tissues allows detecting greater antioxidant capacity; in contrast, the DPPH assay is the most effective for hydrophobic systems.

Phenolic compounds are powerful compounds that counteract stress induced by reactive oxygen species (ROS) in plants, and there is usually a positive correlation between phenolic content and antioxidant activity [48]. The ABTS and DPPH methodologies were used here to assess the antioxidant activity. Measured by both methods, the antioxidant activity was higher in wild than in GCE plants. According to the DPPH and ABTS assays, this activity was in the 8.03–8.67 and 6.49–6.81 mmol TE 100 g^−1^ dw ranges for wild samples and in the 4.39–7.44 and 4.33–6.28 mmol TE 100 g^−1^ dw ranges for the GCE ones, respectively. The higher activity of the wild over GCE plants could be because wild plants are exposed to environmentally stressful factors (sunlight, temperature changes, etc.), which stimulate the synthesis of antioxidant compounds as a defense mechanism. High-salinity soils, such as the ones designed in the various CE trials performed in this work, induce the production of ROS in plants through osmotic stress. Following this, the plants produce antioxidant molecules to counteract such harmful compounds, which help them adapt to high-salinity soils [49].

### 3.4. Phenolic Compounds

Wild plants showed high amounts of TPC in the 503.8 (WM)—575.4 (WT) mg GAE 100 g^−1^ fw range, and TFC were between 191.2 (WM) and 336.8 (WT) mg QE 100 g^−1^ fw (Table 1). These figures were higher than those shown by GCE plants, which displayed TPC amounts in the 395.2 (L3)—455.2 (C3) mg GAE 100 g^−1^ fw range, while TFC values were between 68.0 (L3) and 205.4 (C3) mg QE 100 g^−1^ fw. These values were much higher than the figures assessed through the sum of the individual phenolics using the HPLC–DAD system (Table 2), according to which TPC ranged from 6.4 (L4) to 110.7 (WT) mg 100 g^−1^ fw and TFC were between 6.0 (L4) and 98.4 (WT) mg 100 g^−1^ fw. As expected, all wild plants had TPC and TFC figures significantly higher than those of the GCE samples due to reasons explained in Section 3.3. Moreover, comparing the results for TPC amounts through the F–C method and the HPLC–DAD methodology, an overestimation of the TPC is noted. This is due to the involvement of the non-phenolic reducing agents in the plant extracts, i.e., reducing sugars and certain amino acids, when reducing the F–C reagent [50].

Concerning the phenolic profiles, apigenin and its glucosides were the predominant compounds, representing more than 90% of phenolics in all cases, especially the glucoside-derived apigetrin, the main phenolic found in all samples, especially in the wild specimens. Isoquercetin was also relatively abundant, especially in wild plants. Other minor flavonoids occurring in plants were the glucosides of isorhamnetin and kaempferol and the aglycones luteolin and naringenin. Phenolic acids were minor components within all phenolic profiles, accounting for ~2–10% of TPC in all cases.

The phenolic profiles described here agree with previous reports on the flavonoids of this species by Marzouk et al. (2010) [51] and Al-Jaber (2011) [11], who found, by chromatography column and TLC, respectively, apigenin and its glucosides as the main phenolics in the ethanol extract of the aerial parts of this plant; other phenols included kaempferol and luteolin, both as aglycones and as glucosides.

Interestingly, wild *S. irio* samples contained a higher apigetrin percentage (62.4–78.4% of TPC) than GCE plants (30.6–54.1% of TPC). This flavonoid offers notable health benefits. Its strong antioxidant and anti-inflammatory effects protect cells by reducing oxidative stress and inflammatory markers, like TNF-α and IL-6 [52]. Its cardioprotective benefits include lipid regulation and endothelial cell protection, thus reducing cardiovascular risk [53]. Apigetrin also inhibits gastric cancer progression by inducing apoptosis and regulating the ROS-modulated STAT3/JAK2 pathway [54]. Its neuroprotective properties may aid against neurodegenerative diseases by inhibiting the oxidative damage in neurons [53].

### 3.5. Glucosinolates Content

In Brassicaceae, GLS analyses are widely performed given the importance of these compounds in human health [55]. The most studied *Sisymbrium* species concerning GLS content is *S. officinale* [56,57,58]. The identification of glucoputranjivin-isopropyl-GLS in this work agrees with the data from Cole (1957) [59] and Đulović et al. (2022) [58], who identified this GLS in two *Sisymbrium* species (*S. officinale* and *S. orientale*). Some researchers report that this type of volatile GLS possesses high antimicrobial activity [60] and is highly related to a spicy taste [61].

Another GLS identified in *S. irio* was glucobrassicin, which belongs to the indole compounds type [62]. This GLS has been typified as chemopreventive and anticarcinogenic [63]. The content of these compounds in vegetables is influenced by crop development conditions, climatic factors, and post-harvest storage conditions [64].

It was found in this work that the higher the EC, the higher the GLS content in plant tissues (Figure 3). This agrees with previous reports, in which salinity has been typified as a factor influencing GLS accumulation. For instance, in rocket plants, salt concentrations higher than 34 mM preserve the aesthetic traits of plants, revealing a significant enrichment in GLS [65].

As for the light spectrum, L3 and L4 lamps, which share a combination of green, red, and blue at ~37, ~35, and ~21%, induced higher GLS concentrations in *S. irio* plants. This situation is very similar to that noted in watercress microleaves (*Nasturtium officinale*), in which higher GLS production was found for plants grown under the combination of 50% green, 35% red, and 15% blue lights (RBG light) [66]. On the other hand, Maina et al. (2021) [67] obtained a higher concentration of glucobrassicin in Brassica (*Brassica carinata* L.) microgreens when growing with fluorescent lamps and with lamps with a white + red spectrum. Other workers, such as Moon et al. (2015) [68] and Maina et al. (2021) [67], found that in Brassica plants, the red spectrum in lamps causes an increase in the genes involved in the biosynthesis of indole GLS (glucobrassicin).

### 3.6. Principal Component Analysis

In Figure 4, it can be noted that all wild samples (WT, WM, WG, and WD) have positive scores for PC1; all samples from the EC experiment (C1–C5) have negative scores for both PC1 and PC2; and all samples from the lighting trial have negative scores for PC2. In this plot, it can be noted how different variables influencing the measured parameters: DPPH, ABTS, apigetrin, TPC, glucobrassicin, and glucoputranjivin, have positive loads for PC1 and PC2. Their position in the plot coincides with other applied variables, i.e., EC and UV, allowing us to predict that the latter influences the former. This observation agrees with the conclusions of the experiments in Brassicaceae reported by Šamec et al. (2021) [69], who found that salinity increases the phenolics and glucosinolates contents. On the other hand, in line with the results of this work, it has been reported that UV light differentially tailors glucosinolate and phenolic profiles in Brassicaceae, such as broccoli sprouts [70]. TPC, glucosinolates, DPPH, and ABTS form a group in the plot, thus indicating that the antioxidant activity was mainly exercised by TPC and glucosinolates. Wild samples, which are close in the plot to all these variables, are rich in TPC and glucosinolates due to the high EC and UV in the environment where they develop and, thus, display high antioxidant activity.

It can be noticed that apigenin, red, and far red have similar loads in the plot for PC1 (positive) and PC2 (negative). Although it has been reported that light quality might be associated with polyphenol biosynthesis in higher plants, the effect of light on polyphenol production remains poorly understood (e.g., [71]). However, red and blue light have been reported as favorable for polyphenol biosynthesis in some plants [72]; thus, this situation merits further investigation.

All EC samples share similar scores (PC1 negative, PC2 positive) in the graph along with the variable vitamin C and the blue and green lights. All EC plants were grown under L1 light, located near this cluster. This situation suggests that plants under blue, green, or white light produced higher amounts of vitamin C. In this regard, it has been demonstrated that blue LED light irradiation enhances ascorbic acid content while reducing reactive oxygen species accumulation in Chinese cabbage seedlings [73].

Samples from the lighting experiment (L1–L4) are roughly included in the quadrant of the plot for which both PC1 and PC2 have negative values, and none of the variables are included in this plot area. This means that the application of any of the checked lamps lacks the tendency to induce any biocompound synthesis or bioactivity development.

### 3.7. Antiproliferative Activity of the Ethanol Extracts of S. irio on HT-29 Cancer Cells

After 48 and 72 h of treatment, the MTT assay revealed concentration- and time-dependent inhibitory effects on HT-29 cells for all assayed extracts (Figure 5A,B). The doses of extracts that inhibited cell growth by 50% (GI_50_) are depicted in Figure 5C. After 72 h of cell culture, much better cell-growth inhibition was observed in cells treated with the extracts from wild plants, especially by those from WD and WT (GI_50_ at 72 h of 210 and 330 µg mL^−1^). This situation was expected since the phenolic fraction of wild *S. irio* plants was mainly composed of apigetrin, which exhibits high antitumor activity. The role of apigetrin in cancer has drawn attention due to its potential to target cancer cells selectively while sparing normal cells. This flavonoid has better solubility and stability than other flavonoids; thus, it is expected it could exhibit a strong anticancer effect [74]. In vitro studies on cancer cell lines indicate that apigetrin exerts multiple antitumor effects; for instance, apigetrin promotes TNFα-induced apoptosis, necroptosis, G2/M phase cell cycle arrest, and ROS generation through inhibition of the NF-ΚB pathway in Hep3B liver cancer cells [75]; it inhibits gastric cancer progression by inducing apoptosis and regulating the ROS-modulated STAT3/JAK2 pathway [54]; and it promotes cell apoptosis through the PTEN/PI3K/AKT pathway and inhibits cell migration in cervical cancer HeLa cells [76]. However, besides the high content of apigetrin in *S. irio* leaves, its antitumor activity has been barely tested. The methanol extract of *S. irio* leaves has been assayed against lymphoma cells (U937) through the fluorometric microculture cytotoxicity assay, and a 25–30% inhibition was obtained at 100 μg mL^−1^ [77].

The ethanolic plant extracts richest in GLS obtained in the lighting experiment (L3 and L4) were also tested against colon cancer cells. The GLS contained in the extract were glucoputranjivin and glucobrassicin. The last compound has shown potential anticancer effects through its breakdown products, primarily indole-3-carbinol (I3C) and 3,3′-diindolylmethane (DIM). These compounds influence colon cancer cells through multiple mechanisms, impacting cell growth, apoptosis, and cell cycle regulation (e.g., [78]). Concerning glucoputranjivin, it has been tested on cell lines at a maximum concentration of 100 µg mL^−1^, and the cell viability after 72 h of incubation was 53.18% for MDA-MB-231 (breast cancer cell line), 56.61% for A549 (adenocarcinomic human alveolar basal epithelial cancer cell line), and 60.02% for T24 (human urinary bladder cancer cell line) [58]. Therefore, the observed antiproliferative activity of the *S. irio* ethanol extract on HT-29 cells was likely affected through a synergy involving both flavonoids and GLS.

HT-29 cells are typified as having low sensitivity to phenolics (e.g., [79]). The SI values of HT-29 vs. CCD-18 normal cells were evaluated for samples with GI_50_ < 400 µg mL^−1^ (extracts from wild plants). Extracts at SI > 2 are selective against cancer cells, but the ones showing SI < 2 develop toxicity in normal cells [80]. Thus, the SI value is decisive in evaluating further anticancer activity of any plant material. The SI at 72 h ranged from 2.8 (WM) to 3.1 (WT), which means that the extracts from *S. irio* were selective against HT-29 cells. Future research should focus on purifying phenolics and GLS from *S. irio* to evaluate their antitumor properties separately.

## 4. Materials and Methods

### 4.1. Solvents and Reagents

Unless otherwise specified, all solvents and reagents used in this study were purchased from Merck (Madrid, Spain).

### 4.2. Samples and Growth Conditions

Location data on wild and cultured plants are detailed in Table 3. Two independent experiments were carried out. The first one used five treatments with different electrical conductivities (EC) of the nutrient solution: 2.0 (C1), 2.5 (C2), 3.0 (C3), 3.5 (C4), and 4.0 (C5) dS m^−1^. The second one used different lighting conditions at the same intensity, in which four different light spectra were assayed (Supplementary Figure S3). In both experiments, the plants were grown in a soilless culture system with LED lamps at the University of Almería in controlled growth chambers (10 × 2.5 m) from May to July 2023. The photo-periods were 16/8 h (day/night) at a temperature of 20–22 °C, relative humidity of 80–85%, and photosynthetic photon flux density (400–700 nm) of 250 μmol^−2^ s^−1^. Growth, fertigation, and lighting conditions applied to cultivated plants are detailed in Supplementary File S1 and Supplementary Tables S5 and S6. The pH of the nutrient solutions for both trials was maintained at 5.8 with the addition of nitric acid. Figure 1 shows water (a), potassium (b), and nitrate (c) uptake and fresh (FW) and dry weight (DW) (d) of *S. irio* grown under a soilless culture system under different ECs of nutrient solutions. Figure 2 shows water (a), potassium (b), and nitrate (c) uptake and fresh (FW) and dry weight (DW) (d) of *S. irio* grown under a soilless culture system under different spectra of illumination.

Wild samples were collected after the radiation environment of the sampling sites was characterized by measuring spectral irradiance (Macam SR9910, Macam Photometrics Ltd., Livingston, Scotland, UK), and the photosynthetic photon flux density (PFD) received by the samples at the time of sampling was recorded using a quantum sensor (LI-190SA, LI-COR, Lincoln, NE, USA) (Supplemental File S1). After collecting, samples were labeled, weighed, measured, and placed in a glass desiccator until further analysis. Plant leaves (2 g) were placed in a forced air oven at 105 °C until constant weight to determine the moisture content [81].

### 4.3. Extraction and Quantification of Vitamin C

The analysis of vitamin C (L-ascorbic acid) was performed following the procedure outlined by Volden et al. (2009) [82] with minor modifications (see Supplementary File S1). Vitamin C levels were analyzed using a Finnigan Surveyor HPLC system (Thermo Finnigan, San Jose, CA, USA) equipped with a diode-array detector (DAD) and a reverse-phase column (Luna^®^ Omega, 250 mm × 4.6 mm i.d., 3 µm particle size, from Phenomenex, Torrance, CA, USA). Quantification was performed via external calibration, and the results are expressed as milligrams of ascorbic acid per 100 g of fresh weight (fw).

### 4.4. Extraction of Phenolic Compounds

This methodology is fully described in Supplementary File S1. The extraction of phenolic compounds from *S. irio* leaves was accomplished using ethanol–water (96:4, *v*/*v*), as reported by Lyashenko et al. (2021) [79].

### 4.5. Determination of Total Phenolic and Total Flavonoids Content

The total phenolic content (TPC) was determined using the Folin–Ciocalteu (F–C) assay [83] with minor modifications. The full methodology is detailed in Supplementary File S1. Results are expressed as mg of gallic acid equivalents (GAE) per 100 g of fw using a standard curve of gallic acid. The total flavonoid content (TFC) was analyzed following the method of Zou et al. (2004) [84], as described in Supplementary File S1, and results are given as mg of quercetin equivalents (QE) per 100 g of fw.

### 4.6. Characterization of Phenolic Compounds by HPLC–DAD

**Figure 6:**
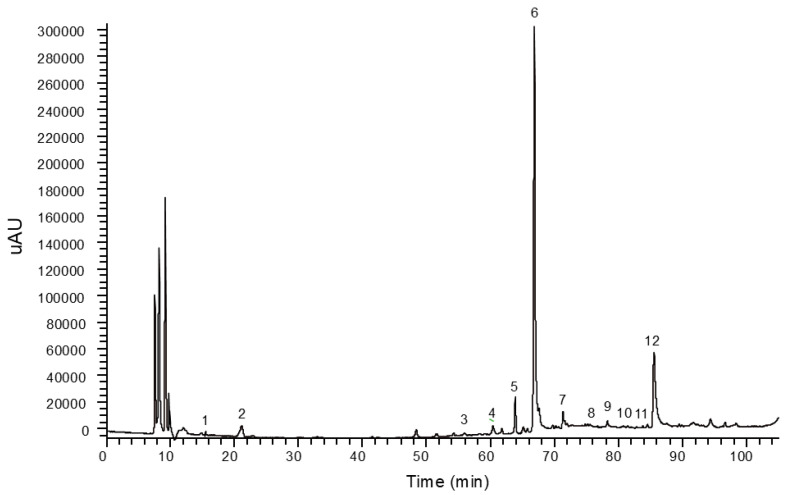
A 254 nm HPLC chromatogram of the phenolic-containing ethanol extract of *S. irio* (sample WG). 1. Gallocatechin (-), 2. Protocatechuic acid, 3. *Trans-p*-coumaric acid, 4. *Trans*-ferulic acid, 5. Quercetin-3-*O*-glucoside, 6. Apigenin-7-*O*-glucoside, 7. Apigenin-7-*O*-rutinoside, 8. Isorhamnetin-3-*O*-glucoside, 9. Luteolin; 10. Kaempferol-3-*O*-rutinoside, 11. Naringenin, 12. Apigenin.

This methodology is detailed in Supplementary File S1. The analyses of phenolic compounds were conducted using the previously described HPLC system. Compounds were separated employing a gradient elution using acidified water (1% acetic acid) (A) and acetonitrile (B) as the mobile phase at 25 °C. The total running time was 105 min. A 254 nm-HPLC–DAD chromatogram of *S. irio* (sample WG) is shown in Figure 6, and the HPLC–DAD parameters for analysis of phenolic-rich extracts of *S. irio* species are detailed in Supplementary Table S3. Quantification of the compounds was accomplished using external calibration curves using pure compounds: chlorogenic acid, *trans-p*-coumaric acid, *trans*-ferulic acid, caffeic acid, luteolin, naringenin, apigenin, apigenin- 7-*O*-glucoside, and quercetin-3-*O*-glucoside.

### 4.7. Characterization of Phenolic Compounds by LC–MS

This methodology is fully detailed in Supplementary File S1, while the LC–MS parameters for analysis of phenolic-containing extracts are detailed in Supplementary Table S4. This table also contains the basis for the structure elucidation of all compounds based on *m*/*z* of the parent molecular ion and fragments. The chromatographic separation was performed on a Thermo Fisher Scientific Transcend 600 LC (Thermo Scientific TranscendTM, Thermo Fisher Scientific, San Jose, CA, USA) using a Hypersil Gold column (250 × 4.6 mm, 5 µm). A flow rate of 0.65 mL min^−1^ was set. The compounds were separated with gradient elution using aqueous acetic acid (acetic acid–H_2_O, 1:99, *v*/*v*) (A) and methanol (B) as eluents at ambient temperature.

The LC system is coupled to a single MS Orbitrap Thermo Fisher Scientific (ExactiveTM, Thermo Fisher Scientific, Bremen, Germany) using an electrospray interface (ESI) (HESI-II, Thermo Fisher Scientific, San Jose, CA, USA) in positive and negative ion mode. The mass spectra were acquired employing alternating acquisition functions: (1) full MS, ESI+, without fragmentation; (2) all-ion fragmentation (AIF), ESI+, with fragmentation (HCD on); (3) full MS, ESI, using the above-mentioned settings; and (4) AIF, ESI, using the settings explained for (2). The mass range in the full scan experiments was set at *m/z* 50–1000. LC chromatograms were acquired using the external calibration mode, which were processed using XcaliburTM version 3.0 with Qualbrowser and Trace Finder 4.0 (Thermo Fisher Scientific, Les Ulis, France). An unknown analysis was carried out with Compound DiscovererTM version 2.1.

### 4.8. Determination of the Antioxidant Activity

Sample extraction was performed as previously described for TPC and TFC analysis, using ethanol–water (96:4, *v*/*v*). This procedure is fully detailed in Supplementary File S1. The assessment of the antioxidant activity by the ABTS method was carried out using the ABTS^•+^ radical solution (2,2′-azinobis (3-ethylbenzothiazoline-6-sulfonic acid)) in ethanol (2.45 mM) [85]. The DPPH method was applied following Skenderidis et al.’s (2018) [86] protocol (Supplementary File S1). Results for ABTS^•+^ and DPPH methods are given as mmol of Trolox Equivalent (TE) 100 g^−1^ dry weight (dw).

### 4.9. Glucosinolates Extraction, HPLC–DAD-ESI/MSn Identification, and HPLC–DAD Quantitation

Identification and quantification were carried out following the method described in Abellán et al. (2021) [87]. After crushing the fresh material, 100 mg of each sample was extracted in 1 mL of ethanol–water (96:4, *v*/*v*) at 70 °C for 20 min with vortex mixing every 5 min. Samples were centrifuged (15,000 g, 15 min) and filtered through 0.22 µm pore size syringe PVDF filters (Análisis Vínicos, Tomelloso, Spain), following the procedure of Velasco et al. (2011) [88]. Samples were stored at 4 °C until further analyses, which were performed in an HPLC-PAD-ESI/MSn Agilent 1200 series, equipped with a photodiode array detector and a serial mass detector (Agilent Technologies, Waldbronn, Germany), using a Kinetex column (5 µm, C18, 100 Å, 150 × 4.6 mm, Phenomenex, Macclesfield, UK). The mobile phase was composed of (A) 1% formic acid and (B) acetonitrile; a gradient was used to obtain 25% B at 25 min and 60% B at 40 min, and then the column was washed out and returned to initial conditions. The flow rate was 0.8 mL min^−1^ and the injection volume was 20 µL. Chromatograms for glucosinolates were recorded at 227 nm. The HPLC system was controlled by ChemStation software (Agilent, version 08.03). Retention times and fragmentation patterns for the identification of glucosinolates are detailed in Supplementary Table S2, and a chromatogram for the identification of GLS by HPLC–DAD is shown in Supplementary Figure S4.

### 4.10. Antitumor Assay

This methodology is detailed in Supplementary File S1. Samples were extracted using ethanol–water (96:4, *v*/*v*), and the antiproliferative activity was evaluated using the MTT assay on the HT-29 human colon cancer cell line and the CCD-18 human colonic myofibroblast cell line, as described by Lyashenko et al. (2021) [79]. The Selectivity Index was calculated as indicated in Supplementary File S1.

### 4.11. Statistical Analysis

All samples were analyzed in triplicate, and means comparison was performed using Duncan’s multiple range test. Data were assessed for normality using a Shapiro–Wilk test and subjected to one-way ANOVA and Principal Component Analysis. Statistical analyses were performed using Statgraphics© Centurion XVI.I (StatPoint Technologies, Warrenton-Virginia, VA, USA).

## 5. Conclusions

The growth response of GCE *S. irio* (fresh weight and dry weight) showed a pattern that is in agreement with the classic production models, and the threshold of decrease in production was close to the optimum yield (fresh and dry) of *S. irio* plants, which was recorded in the 3.0–3.5 dS m^−1^ range. On the other hand, the spectra of lamps designed for use in horticulture significantly influenced the growth of *S. irio* plants. Wild plants exhibited lower vitamin C concentrations than GCE plants, probably due to an absence of stressful factors in the latter plants. However, wild plants contain higher TPC and GLS than GCE ones, and apigetrin, the more outstanding flavonoid occurring in all plants, reached higher percentages in wild plants than in the GCE ones. Two GLS occurred in all the analyzed plants, glucoputranjivin and glucobrassicin, the former at higher percentages than the latter in all cases. As for bioactivity, the wild plants displayed higher antioxidant activity and higher antiproliferative activity against HT-29 cancer cells than the GCE ones, and Principal Component Analysis suggests that EC and UV were responsible for this higher antioxidant activity, in conjunction with the higher TPC and GLS of wild plants over the GCE ones.

Further research should be devoted to fine-tuning the optimum light and EC to be applied to GCE plants to improve their phytochemicals and bioactivities and to studying these in both wild and GCE plants. Given its richness in bioactive compounds and antitumor activities, this green has potential use as a functional food. Still, it is advisable not to consume it excessively since the bioactive compound content of *S. irio* is not yet fully known.

## Figures and Tables

**Table 1:** Moisture, antioxidant activity, vitamin C, and phenolic compounds in *Sisymbrium irio* samples ^1,2,3,4^.

		Antioxidant Activity				
Samples/Codes	Moisture g 100 g^−1^	DPPH mmol TE 100 g^−1^ dw	ABTS mmol TE 100 g^−1^ dw	Vitamin C mg 100 g^−1^ fw	TPC mg GAE 100 g^−1^ fw	TFC mg QE 100 g^−1^ fw
Wild plants						
WD	82.6 ± 0.1 ^bc^	8.03 ± 0.3 ^c^	6.58 ± 0.0 ^b^	75.5 ± 0.4 ^gh^	518.4 ± 4.0 ^b^	328.1 ± 8.6 ^a^
WG	86.1 ± 0.6 ^a^	8.67 ± 0.4 ^a^	6.81 ± 0.2 ^a^	50.7 ± 0.9 ^h^	561.9 ± 23.2 ^a^	204.5 ± 24.2 ^b^
WM	86.0 ± 0.2 ^a^	8.27 ± 0.2 ^bc^	6.54 ± 0.4 ^b^	65.2 ± 0.6 ^gh^	503.8 ± 29.3 ^b^	191.2 ± 32.9 ^bc^
WT	86.0 ± 0.1 ^a^	8.56 ± 0.2 ^ab^	6.49 ± 0.0 ^bc^	84.3 ± 3.0 ^fg^	575.4 ± 37.7 ^a^	336.8 ± 7.1 ^a^
Growth in controlled environment plants						
Saline treatments						
C1 (2.0 dS m^−1^)	80.5 ± 0.7 ^d^	6.14 ± 0.3 ^f^	4.45 ± 0.0 ^g^	95.3 ± 2.7 ^efg^	395.2 ± 4.4 ^ef^	135.7 ± 10.9 ^bcde^
C2 (2.5 dS m^−1^)	81.3 ± 0.5 ^cd^	6.36 ± 0.1 ^ef^	4.81 ± 0.1 ^f^	156.9 ± 15.8 ^bc^	423.7 ± 18.4 ^cd^	128.2 ± 3.2 ^bcde^
C3 (3.0 dS m^−1^)	82.4 ± 1.6 ^bc^	6.49 ± 0.3 ^e^	5.28 ± 0.0 ^e^	193.2 ± 32.2 ^a^	455.2 ± 34.0 ^c^	205.4 ± 24.2 ^b^
C4 (3.5 dS m^−1^)	83.0 ± 0.4 ^b^	6.51 ± 0.2 ^e^	5.70 ± 0.1 ^d^	143.7 ± 20.7 ^cd^	408.2 ± 2.7 ^de^	122.3 ± 32.9 ^cde^
C5 (4.0 dS m^−1^)	80.1 ± 1.8 ^d^	7.25 ± 0.4 ^d^	6.14 ± 0.5 ^c^	186.9 ± 33.9 ^ab^	409.2 ± 10.6 ^de^	132.1 ± 8.9 ^bcde^
Light treatments						
L1 (L18 T8)	81.5 ± 0.5 ^bcd^	7.44 ± 0.2 ^d^	5.62 ± 0.3 ^d^	84.5 ± 3.0 ^fg^	385.3 ± 2.0 ^f^	85.6 ± 0.4 ^de^
L2 (L18 AP67)	82.4 ± 0.2 ^bc^	5.10 ± 0.2 ^g^	4.98 ± 0.0 ^f^	113.9 ± 6.9 ^def^	265.9 ± 7.8 ^g^	72.7 ± 4.0 ^e^
L3 (L18 NS1)	80.3 ± 0.3 ^d^	4.39 ± 0.1 ^h^	4.33 ± 0.4 ^g^	119.4 ± 6.6 ^de^	235.3 ± 5.6 ^g^	68.0 ± 1.2 ^e^
L4 (L18 NS12)	80.6 ± 0.4 ^d^	4.50 ± 0.0 ^h^	6.28 ± 0.2 ^c^	86.2 ± 12.2 ^efg^	250.7 ± 8.3 ^g^	156.7 ± 0.3 ^bcd^
α-tocoferol	-	17.6 ± 0.6	8.8 ± 0.5	-	-	-
Ascorbic acid	-	23.4 ± 1.4	10.5 ± 0.2	-	-	-
Caffeic acid	-	22.2 ± 0.4	10.4 ± 0.3	-	-	-

^1^ Data represent means ± standard deviation of samples analyzed in triplicate. ^2^ Differences in moisture, antioxidant activity, vitamin C, and phenolic compounds in various samples were tested using one-way ANOVA followed by Duncan’s multiple range test. ^3^ Within a column, means followed by different letters are significantly different at *p* < 0.05. ^4^ Results expressed as Gallic Acid Equivalents (GAEs), Quercetin Equivalents (QEs), and Trolox Equivalents (TEs).

**Table 2:** Phenolic compounds in the ethanol extracts of *S. irio* samples (mg 100 g^−1^ fw) ^1,2,3^.

Species/ Codes	Gallocatechin (−) ^4^	Protocatechuic Acid	Trans-p-Coumaric Acid	Trans-Ferulic Acid	Quercetin-3-O-Glucoside ^5^ (Isoquercetin)	Apigenin-7-O-Glucoside (Apigetrin)	Apigenin-7-O-Rutinoside ^6^ (Isorhoifolin)
Wild plants							
WD	n.d.	n.d.	0.2 ± 0.0 ^ab^	1.1 ± 0.2 ^cd^	1.4 ± 0.3 ^cdef^	38.9 ± 1.1 ^c^	1.2 ± 0.3 ^bc^
WG	0.1 ± 0.0 ^cd^	1.5 ± 0.3 ^a^	0.3 ± 0.1 ^ab^	1.0 ± 0.3 ^cd^	5.2 ± 0.9 ^a^	43.2 ± 3.5 ^c^	1.5 ± 0.2 ^b^
WM	0.1 ± 0.0 ^cd^	0.9 ± 0.2 ^b^	0.4 ± 0.2 ^a^	3.7 ± 0.4 ^b^	3.7 ± 0.3 ^b^	57.0 ± 2.9 ^b^	3.2 ± 0.4 ^a^
WT	0.1 ± 0.0 ^cd^	n.d.	0.3 ± 0.1 ^ab^	11.8 ± 1.9 ^a^	2.1 ± 0.2 ^c^	86.8 ± 6.4 ^a^	3.6 ± 0.7 ^a^
Growth in controlled environment plants							
Saline treatments							
C1	0.5 ± 0.2 ^a^	n.d.	0.1 ± 0.0 ^b^	n.d.	0.7 ± 0.2 ^e^	5.9 ± 0.9 ^de^	0.1 ± 0.0 ^f^
C2	0.3 ± 0.1 ^ab^	n.d.	0.1 ± 0.0 ^b^	0.2 ± 0.0 ^d^	0.9 ± 0.1 ^ef^	6.9 ± 0.5 ^de^	0.9 ± 0.2 ^cd^
C3	n.d.	n.d.	0.2 ± 0.0 ^ab^	0.4 ± 0.0 ^d^	1.0 ± 0.3 ^ef^	5.4 ± 1.1 ^de^	0.6 ± 0.2 ^def^
C4	0.1 ± 0.0 ^cd^	n.d.	0.3 ± 0.0 ^ab^	0.7 ± 0.1 ^cd^	1.5 ± 0.4 ^cde^	4.9 ± 0.7 ^de^	0.5 ± 0.1 ^def^
C5	0.2 ± 0.0 ^bc^	n.d.	0.4 ± 0.2 ^a^	1.8 ± 0.3 ^c^	1.8 ± 0.3 ^cd^	7.7 ± 0.8 ^de^	0.8 ± 0.2 ^cde^
Light treatments							
L1	n.d.	n.d.	0.2 ± 0.0 ^ab^	0.2 ± 0.0 ^d^	0.9 ± 0.1 ^ef^	8.0 ± 0.9 ^de^	0.3 ± 0.1 ^def^
L2	n.d.	n.d.	0.1 ± 0.0 ^b^	0.3 ± 0.1 ^d^	0.8 ± 0.2 ^ef^	7.7 ± 1.1 ^de^	0.2 ± 0.0 ^ef^
L3	n.d.	n.d.	0.1 ± 0.0 ^b^	n.d.	1.1 ± 0.3 ^def^	9.0 ± 0.8 ^d^	0.2 ± 0.0 ^ef^
L4	n.d.	n.d.	0.1 ± 0.0 ^b^	0.3 ± 0.1 ^d^	0.7 ± 0.2 ^e^	3.1 ± 0.7 ^e^	0.1 ± 0.0 ^f^
Species/ codes	Isorhamnetin- 3-O-glucoside ^7^	Luteolin	Kaempferol-3-O-rutinoside ^8^ (Nicotiflorin)	Naringenin	Apigenin	Total flavonoids	Total phenolics
Wild plants							
WD	0.1 ± 0.0 ^bc^	1.2 ± 0.2 ^a^	0.1 ± 0.0 ^ab^	0.1 ± 0.0 ^ab^	8.6 ± 0.8 ^d^	51.6 ± 1.4 ^d^	52.9 ± 1.5 ^d^
WG	0.1 ± 0.0 ^bc^	0.5 ± 0.1 ^b^	0.1 ± 0.1 ^ab^	0.2 ± 0.1 ^ab^	15.6 ± 1.1 ^a^	66.2 ± 3.8 ^c^	69.2 ± 3.8 ^c^
WM	0.3 ± 0.1 ^a^	0.9 ± 0.2 ^a^	0.2 ± 0.1 ^a^	0.3 ± 0.1 ^a^	9.4 ± 1.4 ^cd^	75.0 ± 3.3 ^b^	80.1 ± 0.5 ^b^
WT	0.2 ± 0.1 ^ab^	0.5 ± 0.1 ^b^	0.1 ± 0.0 ^ab^	0.1 ± 0.0 ^ab^	5.0 ± 0.4 ^ef^	98.4 ± 6.5 ^a^	110.7 ± 6.9 ^a^
Growth in controlled environment plants							
Saline treatments							
C1	0.1 ± 0.1 ^bc^	0.1 ± 0.0 ^cd^	0.1 ± 0.0 ^ab^	0.1 ± 0.0 ^ab^	3.2 ± 0.5 ^fg^	10.3 ± 1.1 ^ef^	10.9 ± 1.2 ^f^
C2	0.3 ± 0.1 ^a^	0.3 ± 0.1 ^bcd^	n.d.	0.2 ± 0.1 ^ab^	4.9 ± 0.7 ^ef^	14.4 ± 0.9 ^e^	15.0 ± 1.1 ^ef^
C3	n.d.	0.3 ± 0.1 ^bcd^	n.d.	0.2 ± 0.1 ^ab^	3.0 ± 0.8 ^fg^	10.5 ± 1.4 ^ef^	11.1 ± 1.4 ^f^
C4	n.d.	0.4 ± 0.2 ^bc^	0.3 ± 0.2 ^a^	0.1 ± 0.0 ^ab^	7.2 ± 0.8 ^de^	14.9 ± 1.2 ^e^	16.0 ± 1.2 ^e^
C5	n.d.	0.5 ± 0.1 ^b^	n.d.	0.3 ± 0.1 ^a^	9.8 ± 1.5 ^cd^	20.9 ± 1.7 ^d^	23.3 ± 1.8 ^d^
Light treatments							
L1	0.1 ± 0.0 ^bc^	0.1 ± 0.0 ^cd^	n.d.	0.2 ± 0.1 ^ab^	11.9 ± 0.9 ^bc^	21.5 ± 1.3 ^d^	21.9 ± 1.3 ^d^
L2	0.2 ± 0.1 ^ab^	0.2 ± 0.1 ^bcd^	0.1 ± 0.0 ^ab^	n.d.	14.3 ± 1.8 ^ab^	23.5 ± 2.1 ^d^	23.9 ± 2.1 ^d^
L3	n.d.	n.d.	n.d.	0.2 ± 0.1 ^ab^	13.1 ± 2.6 ^ab^	23.6 ± 2.6 ^d^	23.7 ± 2.6 ^d^
L4	n.d.	n.d.	0.1 ± 0.0 ^ab^	0.1 ± 0.1 ^ab^	1.9 ± 0.3 ^g^	6.0 ± 0.4 ^f^	6.4 ± 0.5 ^g^

^1^ Data represent means ± SD of samples analyzed in triplicate; ^2^ Differences in the amounts of phenolic compounds were tested using one-way ANOVA followed by Duncan’s test. ^3^ In a column, means followed by different superscript letters are significantly different at *p* < 0.05; capital letters represent the ANOVA test for means obtained for each species through different samples; ^4^ Gallic acid equivalents; ^5,6,7,8^ Apigenin-7-*O*-glucoside equivalents; n.d.: not detected.

**Table 3:** Data on plant status, harvesting locations, and cultivation conditions (electrical conductivities of the nutrient solutions and lamp types) of the *Sisymbrium irio* samples analyzed in this work.

Sample/Code	Status	Location/ Geographical Coordinates	Conductivity of the Nutrient Solution/Soil (dS m^−1^)	Lamp Type
WD	Wild	Aguadulce, Almería (36.814444 −2.571666)	2.9	-
WG	Wild	Algeciras, Cádiz (36.105488 −5.452843)	2.1	-
WM	Wild	Almería, Almería (36.838140 −2.459740)	4.4	-
WT	Wild	El Toyo, Almería (36.837729 −2.327154)	5.6	-
C1	GCE ^a^	Growth chamber, UAL ^b^ (36.831919 −2.402191)	2.0	L18 T8 Roblan^®^
C2	GCE	Growth chamber, UAL (36.831919 −2.402191)	2.5	L18 T8 Roblan^®^
C3	GCE	Growth chamber, UAL (36.831919 −2.402191)	3.0	L18 T8 Roblan^®^
C4	GCE	Growth chamber, UAL (36.831919 −2.402191)	3.5	L18 T8 Roblan^®^
C5	GCE	Growth chamber, UAL (36.831919 −2.402191)	4.0	L18 T8 Roblan^®^
L1	GCE	Growth chamber, UAL (36.831919 −2.402191)	2.0	L18 T8 Roblan^®^
L2	GCE	Growth chamber, UAL (36.831919 −2.402191)	2.0	L18 AP67 Valoya^®^
L3	GCE	Growth chamber, UAL (36.831919 −2.402191)	2.0	L18 NS1 Valoya^®^
L4	GCE	Growth chamber, UAL (36.831919 −2.402191)	2.0	L18 NS12 Valoya^®^

^a^ Grown in a Controlled Environment; ^b^ University of Almería.

## Data Availability

All data concerning this research are available in the figures and tables of the article.

## Supplementary Materials

The following supporting information can be downloaded at https://www.mdpi.com/article/10.3390/molecules30010031/s1: Supplementary File S1. Material and Methods. Supplementary figures: Supplementary Figure S1. Electrical conductivity (EC); Supplementary Figure S2. EC and pH of drainage versus spectrum; Supplementary Figure S3. Spectrum of LED lamps; Supplementary Figure S4. HPLC chromatogram of *S. irio* glucosinolates. Supplementary tables: Supplementary Table S1. Phenolic compound occurrence by LC–MS; Supplementary Table S2. Parameters of LED; Supplementary Table S3. Glucosinolate compound identification by HPLC–MS; Supplementary Table S4. Nutrient solution; Supplementary Table S5. Chromatography parameters HPLC–DAD; Supplementary Table S6. Phenolic parameters of LC–MS and identification basis. References [89,90,91,92,93,94,95,96,97,98,99] are cited in the Supplementary Materials.

## References

[1] Carvalho A.M., Barata A.M. (2016). The consumption of wild edible plants. Wild Plants, Mushrooms and Nuts: Functional Food Properties and Applications.

[2] Vohora S.B., Nagvi S.A., Kumar H.I. (1980). Antipyretic analgesic and antimicrobial studies on *Sismbruym irio*. Planta Med..

[3] Tiwari M., Bhargava P. (2021). Current updates on *Sisymbrium irio* linn: A traditional medicinal plant. Plant Arch..

[4] Guil-Guerrero J.L., Giménez-Martínez J.J., Torija-Isasa M.E. (1999). Nutritional composition of wild edible crucifer species. J. Food Biochem..

[5] Khan M.S.Y., Javed K., Hasnain Khan M. (1991). Chemical constituents of the aerial parts of *Sisymbrium irio*. J. Indian Chem. Soc..

[6] Itziar A., Marii A., Peromartino Z. (1982). Nine acetylated glycosides of *Sismbrium gilliesii*. Phytochemistry.

[7] Rizk A., Heiba H.I., Mayerigi H.A., Batanony K.H. (1986). Phytochemical screening of some *Sismbrium* species. Fitoterapia.

[8] Lockwood G.B., Fsharypuor S. (1986). Some chemical constituents of *Sisymbrium sophia*. Bull. Liaison Group Polyphenol.

[9] Arayno M.S., Zafor N. (1983). Phenolic compounds of *Sisymbrium incisum*. J. Pharm..

[10] Krets L.G., Domashenko L.G., Negare N.P., Railyan A.F. (1987). Glycoside from *Sisymbrium* species. Ser. Biol. Khim. Nauk.

[11] Al-Jaber N.A. (2011). Phytochemical and biological studies of *Sisymbrium irio* L. growing in Saudi Arabia. J. Saudi Chem. Soc..

[12] Gruenwald J., Brendler T., Jaenicke C. (2004). Hedge mustard-*Sisymbrium officinale*. PDR for Herbal Medicines.

[13] Melrose J. (2019). The glucosinolates: A sulphur glucoside family of mustard anti-tumour and antimicrobial phytochemicals of potential therapeutic application. Biomedicines.

[14] Nájera C., Ros M., Moreno D.A., Hernández-Lara A., Pascual J.A. (2024). Combined effect of an agro-industrial compost and light spectra composition on yield and phytochemical profile in mizuna and pak choi microgreens. Heliyon.

[15] Simsek M., Whitney K. (2024). Examination of primary and secondary metabolites associated with a plant-based diet and their impact on human health. Foods.

[16] Akram M., Jabeen F., Riaz M., Khan F.S., Okushanova E., Imran M., Ali S.M., Riaz T., Egbuna C., Ezeofor N.J. (2021). Health benefits of glucosinolate isolated from cruciferous and other vegetables. Preparation of Phytopharmaceuticals for the Management of Disorders.

[17] Maher S., Nisar S., Aslam S.M., Saleem F., Behlil F., Imran M., Aslam P. (2023). Synthesis and characterization of ZnO nanoparticles derived from biomass (*Sisymbrium irio*) and assessment of potential anticancer activity. ACS Omega.

[18] Al-Massarani S.M., El Gamal A.A., Alam P., Al-Sheddi E.S., Al-Oqail M.M., Farshori N.N. (2017). Isolation, biological evaluation and validated HPTLC-quantification of the marker constituent of the edible Saudi plant *Sisymbrium irio* L. Saudi Pharm. J..

[19] Delian E., Chira A., Bădulescu L., Chira L. (2015). Insights into microgreens physiology. Sci. Pap.-Ser. B Hortic..

[20] Ferrón-Carrillo F., Guil-Guerrero J.L., González-Fernández M.J., Lyashenko S., Battafarano F., Cunha-Chiamolera T.P.L., Urrestarazu M. (2021). LED enhances plant performance and both carotenoids and nitrates profiles in lettuce. Plant Food Hum. Nutr..

[21] Maas E.V., Hoffman G.J. (1977). Crop salt tolerance-current assessment. J. Irrig. Drain. Div..

[22] Chourak Y., Belarbi E.H., Cunha-Chiamolera T.P.L., Guil-Guerrero J.L., Carrasco G., Urrestarazu M. (2022). Effect of macro-nutrient conditions and electrical conductivity on the quality of saffron grown in soilless culture systems. J. Soil Sci. Plant Nutr..

[23] Moya C., Oyanedel E., Verdugo G., Flores M.F., Urrestarazu M., Álvaro J.E. (2017). Increased electrical conductivity in nutrient solution management enhances dietary and organoleptic qualities in soilless culture tomato. HortScience.

[24] Cuartero J., Fernández R. (1999). Tomato and salinity. Sci. Hortic..

[25] Signore A., Serio F., Santamaria P. (2016). A targeted management of the nutrient solution in a soilless tomato crop according to plant needs. Front. Plant Sci..

[26] Avercheva O.V., Berkovich Y.A., Erokhin A.N., Zhigalova T.V., Pogosyan S.I., Smolyanina S.O. (2009). Growth and photosynthesis of Chinese cabbage plants grown under light-emitting diode-based light source. Russ. J. Plant Physiol..

[27] Zhang X., Bian Z., Yuan X., Chen X., Lu C. (2020). A review on the effects of light-emitting diode (LED) light on the nutrients of sprouts and microgreens. Trends Food Sci. Technol..

[28] Morrow R.C. (2008). LED lighting in horticulture. HortScience.

[29] Ying Q., Kong Y., Jones-Baumgardt C., Zheng Y. (2020). Responses of yield and appearance quality of four brassicaceae microgreens to varied blue light proportion in red and blue light-emitting diodes lighting. Sci. Hortic..

[30] Cunha-Chiamolera T.P.L., Chileh-Chelh T., Urrestarazu M., Ezzaitouni M., López-Ruiz R., Gallón-Bedoya M., Rincón-Cervera M.Á., Guil-Guerrero J.L. (2024). Crop productivity, phytochemicals, and bioactivities of wild and grown in controlled environment slender amaranth (*Amaranthus viridis* L.). Agronomy.

[31] Rincón-Cervera M.Á., Cunha-Chiamolera T.P.L., Chileh-Chelh T., Carmona-Fernández M., Urrestarazu M., Guil-Guerrero J.L. (2024). Growth parameters, phytochemicals, and antitumor activity of wild and cultivated ice plants (*Mesembryanthemum crystallinum* L.). Food Sci. Nutr..

[32] Peçanha D.A., Cunha-Chiamolera T.P.L., Chourak Y., Martínez-Rivera E.Y., Urrestarazu M. (2021). Effect of the matric potential on growth and water, nitrate, and potassium absorption of vegetables under soilless culture. J. Soil Sci. Plant Nutr..

[33] Urrestarazu M., Carrasco G. (2023). Soilless Culture and Hydroponics.

[34] Brendel O. (2021). The relationship between plant growth and water consumption: A history from the classical four elements to modern stable isotopes. Ann. For. Sci..

[35] Sonneveld C., Voogt W. (2009). Plant Nutrition of Greenhouse Crops.

[36] Gallegos-Cedillo V.M., Urrestarazu M., Álvaro J.E. (2016). Influence of salinity on transport of nitrates and potassium by means of the xylem sap content between roots and leaves in young tomato plants. J. Soil Sci. Plant Nutr..

[37] Rodríguez D., Reca J., Martínez J., Lao M.T., Urrestarazu M. (2014). Effect of controlling the leaching fraction on the fertigation and production of a tomato crop under soilless culture. Sci. Hortic..

[38] Adams P. (1994). Nutrition of greenhouse vegetables in NFT and hydroponic systems. Acta Hortic..

[39] Arnon D.I., Johnson C.M. (1942). Influence of hydrogen ion concentration on the growth of higher plants under controlled conditions. Plant Physiol..

[40] Truog E. (1948). Lime in relation to availability of plant nutrients. Soil Sci..

[41] Álvarez-García M., Urrestarazu M., Guil-Guerrero J.L., Jiménez-Becker S. (2019). Effect of fertigation using fish production wastewater on Pelargonium x zonale growth and nutrient content. Agric. Water Manag..

[42] Sonneveld C., Bajaj Y.P.S. (1991). Rockwool as a substrate for greenhouse crops. Biotechnology in Agriculture and Forestry.

[43] Pennisi G., Blasioli S., Cellini A., Maia L., Crepaldi A., Braschi I., Spinelli F., Nicola S., Fernandez J.A., Stanghellini C. (2019). Unraveling the role of red LED lights on resource use efficiency and nutritional properties of indoor grown sweet basil. Front. Plant Sci..

[44] Spalholz H., Perkins-Veazie P., Hernández R. (2020). Impact of sun-simulated white light and varied blue spectrums on the growth, morphology, development, and phytochemical content of green- and red-leaf lettuce at different growth stages. Sci. Hortic..

[45] Castañeda-Loaiza V., Oliveira M., Santos T., Schüler L., Lima A.R., Gama F., Salazar M., Neng N.R., Nogueira J.M.F., Varela J. (2020). Wild vs cultivated halophytes: Nutritional and functional differences. Food Chem..

[46] Guil J.L., Rodríguez-García I., Torija E. (1997). Nutritional and toxic factors in selected wild edible plants. Plant Foods Hum. Nutr..

[47] Floegel A., Kim D.O., Chung S.J., Koo S.I., Chun O.K. (2011). Comparison of ABTS/DPPH assays to measure antioxidant capacity in popular antioxidant-rich US foods. J. Food Compos. Anal..

[48] Calvo M.M., Martín-Diana A.B., Rico D., López-Caballero M.E., Martínez-Álvarez O. (2022). Antioxidant, antihypertensive, hypoglycaemic, and nootropic activity of a polyphenolic extract from the halophyte ice plant (*Mesembryanthemum crystallinum*). Foods.

[49] García-Caparrós P., Hasanuzzaman M., Lao M.T., Hasanuzzaman M., Fotopoulos V., Nahar K., Fujita M. (2019). Oxidative stress and antioxidant defense in plants under salinity. Reactive Oxygen, Nitrogen and Sulfur Species in Plants.

[50] Sadeer N.B., Montesano D., Albrizio S., Zengin G., Mahomoodally M.F. (2020). The versatility of antioxidant assays in food science and safety-Chemistry, applications, strengths, and limitations. Antioxidants.

[51] Marzouk M.M., Al-Nowaihi A.M., Kawashty S.A., Saleh N.A.M. (2010). Chemosystematic studies on certain species of the family Brassicaceae (Cruciferae) in Egypt. Biochem. Syst. Ecol..

[52] Han H., Kim J.E., Lee H.J. (2024). Effect of apigetrin in pseudo-SARS-CoV-2-induced inflammatory and pulmonary fibrosis in vitro model. Sci. Rep..

[53] Ali F., Rahul, Naz F., Jyoti S., Siddique Y.H. (2017). Health functionality of apigenin: A review. Int. J. Food Prop..

[54] Sun Q., Lu N.N., Feng L. (2018). Apigetrin inhibits gastric cancer progression through inducing apoptosis and regulating ROS-modulated STAT3/JAK2 pathway. Biochem. Biophys. Res. Commun..

[55] Possenti M., Baima S., Raffo A., Durazzo A., Giusti A.M., Natella F., Mérillon J.M., Ramawat K. (2016). Glucosinolates in Food. Glucosinolates.

[56] Borgonovo G., Zimbaldi N., Guarise M., Bedussi F., Winnig M., Vennegeerts T., Bassoli A. (2019). Glucosinolates in *Sisymbrium officinale* (L.) Scop.: Comparative analysis in cultivated and wild plants and in vitro assays with T2Rs bitter taste receptors. Molecules.

[57] Zorzan M., Zucca P., Collazuol D., Peddio S., Rescigno A., Pezzani R. (2020). *Sisymbrium officinale*, the plant of singers: A review of its properties and uses. Planta Med..

[58] Đulović A., Popović M., Burčul F., Čikeš Čulić V., Marín A., Ruščić M., Anđelković N., Blažević I. (2022). Glucosinolates of *Sisymbrium officinale* and *S. orientale*. Molecules.

[59] Cole R.A. (1976). Isothiocyanates, nitriles and thiocyanates as products of autolysis of glucosinolates in Cruciferae. Phytochemistry.

[60] Blažević I., Radonić A., Mastelić J., Zekić M., Skočibušić M., Maravić A. (2010). Hedge mustard (*Sisymbrium officinale*): Chemical diversity of volatiles and their antimicrobial activity. Chem. Biodivers..

[61] Sultana T., Savage G.P., McNeil D.L., Porter N.G., Clark B. (2003). Comparison of flavour compounds in wasabi and horseradish. J. Food Agric. Environ..

[62] Sikorska-Zimny K., Beneduce L. (2021). The metabolism of glucosinolates by gut microbiota. Nutrients.

[63] Radošević K., Srček V.G., Bubalo M.C., Brnčić S.R., Takács K., Redovniković I.R. (2017). Assessment of glucosinolates, antioxidative and antiproliferative activity of broccoli and collard extracts. J. Food Compos. Anal..

[64] Deng Q., Zinoviadou K.G., Galanakis C.M., Orlien V., Grimi N., Vorobiev E., Barba F.J. (2015). The effects of conventional and non-conventional processing on glucosinolates and its derived forms, isothiocyanates: Extraction, degradation, and applications. Food Eng. Rev..

[65] Corti E., Falsini S., Gonnelli C., Pieraccini G., Nako B., Papini A. (2023). Salt-affected rocket plants as a possible source of glucosinolates. Int. J. Mol. Sci..

[66] Klimek-Szczykutowicz M., Prokopiuk B., Dziurka K., Pawłowska B., Ekiert H., Szopa A. (2022). The influence of different wavelengths of LED light on the production of glucosinolates and phenolic compounds and the antioxidant potential in in vitro cultures of *Nasturtium officinale* (watercress). Plant Cell Tissue Organ Cult..

[67] Maina S., Ryu D.H., Cho J.Y., Jung D.S., Park J.-E., Nho C.W., Bakari G., Misinzo G., Jung J.H., Yang S.-H. (2021). Exposure to salinity and light spectra regulates glucosinolates, phenolics, and antioxidant capacity of *Brassica carinata* L. microgreens. Antioxidants.

[68] Moon J., Jeong M.J., Lee S.I., Lee J.G., Hwang H., Yu J., Kim J.A. (2015). Effect of LED mixed light conditions on the glucosinolate pathway in Brassica rapa. J. Plant Biotechnol..

[69] Šamec D., Linić I., Salopek-Sondi B. (2021). Salinity stress as an elicitor for phytochemicals and minerals accumulation in selected leafy vegetables of Brassicaceae. Agronomy.

[70] Moreira-Rodríguez M., Nair V., Benavides J., Cisneros-Zevallos L., Jacobo-Velázquez D.A. (2017). UVA, UVB light doses and harvesting time differentially tailor glucosinolates and phenolic profiles in Broccoli Sprouts. Molecules.

[71] Lan H., Wang C., Yang Z., Zhu J., Fang W., Yin Y. (2024). The impact of lighting treatments on the biosynthesis of phenolic acids in black wheat seedlings. Foods.

[72] Ebisawa M., Shoji K., Kato M., Shimomura K., Goto F., Yoshihara T. (2008). Supplementary ultraviolet radiation B together with blue light at night increased quercetin content and flavonol synthase gene expression in leaf lettuce (*Lactuca sativa* L.). Environ. Control Biol..

[73] Kang C.H., Yoon E.K., Muthusamy M., Kim J.A., Jeong M.J., Lee S.I. (2020). Blue LED light irradiation enhances L-ascorbic acid content while reducing reactive oxygen species accumulation in chinese cabbage seedlings. Sci. Hortic..

[74] Smiljkovic M., Stanisavljevic D., Stojkovic D., Petrovic I., Vicentic J.M., Popovic J., Grdadolnik S.G., Markovic D., Sankovic-Babice S., Glamoclija J. (2017). Apigenin-7-*O*-glucoside versus apigenin: Insight into the modes of anticandidal and cytotoxic actions. EXCLI J..

[75] Bhosale P.B., Abusaliya A., Kim H.H., Ha S.E., Park M.Y., Jeong S.H., Vetrivel P., Heo J.D., Kim J.A., Won C.K. (2022). Apigetrin promotes TNFα-Induced apoptosis, necroptosis, G2/M phase cell cycle arrest, and ROS generation through inhibition of NF-κB pathway in Hep3B liver cancer cells. Cells.

[76] Liu M.-M., Ma R.-H., Ni Z.-J., Thakur K., Cespedes-Acuña C.L., Jiang L., Wei Z.-J. (2020). Apigenin 7-*O*-glucoside promotes cell apoptosis through the PTEN/PI3K/AKT pathway and inhibits cell migration in cervical cancer HeLa cells. Food Chem. Toxicol..

[77] El-Seedi H.R., Burman R., Mansour A., Turki Z., Boulos L., Gullbo J., Göransson U. (2013). The traditional medical uses and cytotoxic activities of sixty-one Egyptian plants: Discovery of an active cardiac glycoside from *Urginea maritima*. J. Ethnopharmacol..

[78] Williams D.E. (2021). Indoles derived from glucobrassicin: Cancer chemoprevention by indole-3-carbinol and 3,3′-diindolylmethane. Front. Nutr..

[79] Lyashenko S., Fabrikov D., González-Fernández M.J., Gómez-Mercado F., Ruiz R.L., Fedorov A., Guil-Guerrero J.L. (2021). Phenolic composition and in vitro antiproliferative activity of *Borago* spp. seed extracts on HT-29 cancer cells. Food Biosci..

[80] Vichitsakul K., Laowichuwakonnukul K., Soontornworajit B., Poomipark N., Itharat A., Rotkrua P. (2023). Anti-proliferation and induction of mitochondria-mediated apoptosis by *Garcinia hanburyi* resin in colorectal cancer cells. Heliyon.

[81] Ramos-Bueno R.P., Rincón-Cervera M.A., González-Fernández M.J., Guil-Guerrero J.L. (2016). Phytochemical composition and antitumor activities of new salad greens: Rucola (*Diplotaxis tenuifolia*) and corn salad (*Valerianella locusta*). Plant Foods Hum. Nutr..

[82] Volden J., Bengtsson G.B., Wicklund T. (2009). Glucosinolates, L-ascorbic acid, total phenols, anthocyanins, antioxidant capacities and color in cauliflower (*Brassica oleracea* L. ssp. *botrytis*); effects of long-term freezer storage. Food Chem..

[83] Singleton V.L., Orthofer R., Lamuela-Raventós R.M. (1999). Analysis of total phenols and other oxidation substrates and antioxidants by means of folin-ciocalteu reagent. Methods Enzymol..

[84] Zou Y., Lu Y., Wei D. (2004). Antioxidant activity of flavonoid-rich extracts of *Hypericum perforatum* L. in vitro. J. Agric. Food Chem..

[85] Re R., Pellegrini N., Proteggente A., Pannala A., Yang M., Rice-Evans C. (1999). Antioxidant activity applying an improved ABTS radical cation decolorization assay. Free Radic. Biol. Med..

[86] Skenderidis P., Kerasioti E., Karkanta E., Stagos D., Kouretas D., Petrotos K., Tsakalof A. (2018). Assessment of the antioxidant and antimutagenic activity of extracts from goji berry of Greek cultivation. Toxicol. Rep..

[87] Abellán Á., Domínguez-Perles R., García-Viguera C., Moreno D.A. (2021). In vitro evidence on bioaccessibility of flavonols and cinnamoyl derivatives of cruciferous sprouts. Nutrients.

[88] Velasco P., Francisco M., Moreno D.A., Ferreres F., García-Viguera C., Cartea M.E. (2011). Phytochemical fingerprinting of vegetable *Brassica oleracea* and *Brassica napus* by simultaneous identification of glucosinolates and phenolics. Phytochem. Anal..

[89] Ramos-Bueno R.P., Romero-González R., González-Fernández M.J., Guil-Guerrero J.L. (2017). Phytochemical composition and in vitro anti-tumour activities of selected tomato varieties. J. Sci. Food Agric..

[90] Sonneveld C., Straver N.B. (1994). Nutrient solution for vegetables and flowers grown in water or substrates. Voedingspoloss. Glas..

[91] Rawat P., Singh Y., Tiwari S., Mishra D.K., Kanojiya S. (2023). The characterization and quantification of structures of *Cajanus scarabaeoides* phytochemicals and their seasonal variation analysis using ultra-performance liquid chromatography-tandem mass spectrometry. Rapid Commun. Mass Spectrom..

[92] Dantas C.A.G., Abreu L.S., da Cunha H.N., Veloso C.A.G., Souto A.L., Agra M.F., Costa V.C.O., da Silva M.S., Tavares J.F. (2021). Dereplication of phenolic derivatives of three *Erythroxylum* species using liquid chromatography coupled with ESI-MS^n^ and HRESIMS. Phytochem. Anal..

[93] Marcum C.L., Jarrell T.M., Zhu H., Owen B.C., Haupert L.J., Easton M., Hosseinaei O., Bozell J., Nash J.J., Kenttämaa H.I. (2016). A fundamental tandem mass spectrometry study of the collision activated dissociation of small, deprotonated molecules relaed to lignin. ChemSusChem.

[94] Abrankó L., Szilvássy B. (2015). Mass spectrometric profiling of flavonoid glycoconjugates possessing isomeric aglycones. J. Mass Spectrom..

[95] Liang Y., Zhao W., Wang C., Wang Z., Wang Z., Zhang J. (2018). A comprehensive screening and identification of genistin metabolites in rats based on multiple metabolite templates combined with UHPLC-HRMS analysis. Molecules.

[96] Wu F.P., Liu L.H., Jin P., Pu H., Cai W. (2019). Determination of metabolites of phloretin in rats using UHPLC-LTQ-Orbitrap mass spectrometry. Trop. J. Pharm. Res..

[97] Fabre N., Rustan I., de Hoffmann E., Quetin-Leclercq J. (2001). Determination of flavone, flavonol, and flavanone aglycones by negative ion liquid chromatography electrospray ion trap mass spectrometry. J. Am. Soc. Mass Spectrom..

[98] Yehia S.M., Ayoub I.M., Watanabe M., Devkota H.P., Singab A.N.B. (2023). Metabolic profiling, antioxidant, and enzyme inhibition potential of *Iris pseudacorus* L. from Egypt and Japan: A comparative study. Sci. Rep..

[99] Śliwka-Kaszyńska M., Anusiewicz I., Skurski P. (2022). The mechanism of a retro-diels–alder fragmentation of luteolin: Theoretical studies supported by electrospray ionization tandem mass spectrometry results. Molecules.

