# Supplementary material for: London Rocket (Sisymbrium irio L.) as Healthy Green: Bioactive Compounds and Bioactivity of Plants Grown in Wild and Controlled Environments
Source: Molecules. 2024 Dec 25;30(1):31. doi: 10.3390/molecules30010031 (PMC11721195; doi:10.3390/molecules30010031)
Supplement: Supplementary file 1 [file molecules-30-00031-s001.zip › Supplementary File S1. Material and Methods.pdf]

Supplementary Materials of the article:

London Rocket (*Sisymbrium irio* L.) as Healthy Green: Bioactive Compounds and Bioactivity of Plants Grown in Wild and Controlled Environments Table

Supplementary File S1. Material and Methods

1. Reagents and Chemicals

Unless otherwise indicated, all chemicals and solvents were purchased at high purity grade from Merck (Madrid, Spain). L-Ascorbic acid was procured from Labkem (Barcelona, Spain). An aluminium chloride and sodium carbonate were sourced from Sigma-Aldrich Co. (St Louis, MO, USA). Sodium nitrite, sodium hydroxide, and oxalic acid were acquired from Panreac (Barcelona, Spain). The Folin-Ciocalteu (F-C) reagent, gallic acid, quercetin, DPPH, $ABTS^{+\bullet}$, and Trolox standards were obtained from Sigma-Aldrich Co. (St Louis, MO, USA). Water purification was carried out using a Milli-Q system (Millipore).

2. Growth, fertigation, and lighting conditions applied to S. irio *grown in a controlled environment*

S. irio plants were grown in two independent experiments. In the first one, five treatments were used with different electrical conductivities in the nutrient solution, 2.0 (C1), 2.5 (C2), 3.0 (C3), 3.5 (C4) and 4.0 (C4) dS m^{-1}. A Roblan® L18 T8 white LED lamp (Toledo, Spain) was used for the test. In the second experiment, four LED lamps were evaluated: T8 Roblan® as control (L1), and three specifically designed horticultural lighting fixtures: AP67, NS1 and NS12Valoya® (Helsinki, Finland) as treatment L2, L3 and L4, respectively.

For both experiments, three light-emitting diode (LED) lamps were used, both on a surface of 0.504 m^2. All lamps had the same length and power (18 W). The spectra of each treatment were measured with a UPRtek MK350S LED (UPRtek, Taiwan). LP471-PHOT and LP471-PAR sensors (Delta OHM®, Padua, Italy) were used to

measure luminance (lux) and photosynthetic photon flux, PPF (mmol m^{-2} s^{-1}), as described by Ferrón-Carrillo et al. (2021) [20].

The application of the fertigation was performed when the water in the culture unit reached 10% of the readily available water, and the necessary volume was added to obtain between 15-25% drainage [32,37]. For each treatment, two controls were established as fertigation controls, consisting of a control dripper and a drainage tray that served as measurement and monitoring points for the fertigation supplied and the uptake response. The volume, pH, electrical conductivity (EC), nitrate, and potassium content of the supplied nutrient solution and drainage were measured daily in each pot with a container that was adapted to its morphology, using a Crison MM40+ pH meter (Hach® LPV2500.98.0002, Loveland, CO, USA), an EC-Meter Crison BASIC 30 conductivity meter (Hach® LPV2500.98.0002, Loveland, Colorado, USA), and a LAQUATwin B-742, and B-731 (Horiba®, Moulton, Northampton,UK), respectively. The ratio of drainage was measured using a test tube graduated to one-hundredth of a millimetre.

3. Evaluation of growth parameters

The evaluation of growth parameters was carried out after transplantation. The experimental unit was four plants per treatment and four replicates of each treatment. The plants were divided by their different organs; then, the fresh weight of roots, stems, and leaves was obtained, and the dry weight was calculated by placing the material in an oven (Thermo Scientific Heratherm, Waltham, MA, USA) at 85 °C until achieving a constant weight. Weights were measured using a precision analytical balance (model AX 124/E, OHAUS Corporation, Parsippany, NJ, USA), with an accuracy of four-tenths of a gram.

Plants were collected at the baby leaf stage (16-17 leaves). After harvesting, plants were stored in thermal bags and frozen at -24 ºC until processing. Once in the laboratory, the greens were labelled, weighed, measured, and placed in a glass desiccator until analysis.

4. Wild plants collecting

Wild samples were collected after the radiation environment of the sampling sites was characterized by measuring spectral irradiance between 280 and 700 nm with a spectroradiometer (Macam SR9910, Macam Photometrics Ltd., Livingston, Scotland), placing the sensor close to the plants. Spectra were taken around midday on comparable sunny days prior to or following the collection days. Given that the radiation spectra measured in the different sampling sites were very similar, only the average spectra of

the conditions are shown (Supplementary Table S6). The photosynthetic photon flux density (PFD) received by the samples at the time of sampling was measured using a quantum sensor (LI-190SA, LI-COR, Lincoln, NE, USA). After collecting, samples were labelled, weighed, measured, and placed in a glass desiccator until analysis.

5. Extraction and quantification of vitamin C

Extraction and quantification of vitamin C (ascorbic plus dehydroascorbic acids) was carried out according to previous protocols, with minor modifications [82]. Fresh leaves were minced, and 1 g was collected and extracted with 20 mL of an aqueous oxalic acid solution (1% w/v). The mixture was centrifuged at 2,500 g for 10 min and then filtered to collect the filtrate (extract). The extract was filtered again through a 0.22 µm Millipore filter and 1 mL was used for chromatographic analysis. HPLC analysis of vitamin C was carried out in a Finnigan Surveyor chromatograph equipped with a diode-array detector (DAD) and a reverse-phase C18 column (Luna® Omega, 250 mm x 4.6 mm i.d., 3 µm particle size, Phenomenex, USA). The mobile phase consisted of (A) methanol and (B) water with 0.1% oxalic acid in an isocratic mode 5% A and 95% B at 0.4 mL min^{-1} for 15 min. Detection was performed at 254 nm, and the injection volume was 10 µL. Vitamin C was quantified by external calibration and results were recorded as mg 100 g^{-1} fw. Determinations were carried out in triplicate and all data are reported as mean value ± standard deviation.

6. Extraction of phenolics from S. irio *samples*

Extraction and analysis of phenolic compounds were conducted following the method outlined by Lyashenko et al. (2021) [79], with slight adjustments. Approximately 1 g of the fresh leaves was weighed and then mixed with 10 mL of ethanol:water (96:4, v/v). After maceration, sonication, and centrifugation at 4000 rpm for 10 min, 1 mL of the phenolic extract was filtered through a 0.22 µm membrane filter before chromatographic analysis.

7. Total phenols and total flavonoids

Total phenolic content (TPC) was measured using the Folin-Ciocalteu (F-C) assay with minor modifications [83]. Briefly, 10 µL of phenolic extracts in 0.79 mL of MiliQ water (preparation of the phenolic extract is fully detailed in Supplementary File S1-5), and 50 µL of Folin-Ciocalteu reagent were mixed, vortexed, and allowed to stand for 5 min at room temperature. Next, 150 µL of a 20% sodium carbonate solution was added and vortexed. A control sample was also prepared. After incubation at room temperature for 2 h in darkness, the absorbance of the mixture was read at 765 nm on a UV-VIS

spectrophotometer. Deionized water was used as blank. Results were expressed as mg of gallic acid equivalents (GAE) per 100 g of sample using a standard curve of gallic acid (0-500 mg 100 mL^{-1}). Determinations were carried out in triplicate.

The total flavonoid content (TFC) was determined using the aluminium chloride method with minor modifications [84]. Briefly, 0.5 mL of the phenolic extract was collected and 150 µL of 5% $NaNO_2$ solution was added. After 5 min, 150 µL of 10% $AlCl_3$ solution was added to the mixture, which was kept at room temperature for 5 min, followed by adding 0.7 mL of 1M NaOH. The absorbance was then immediately measured at 510 nm on a UV-VIS spectrophotometer. Deionized water was used as blank. The results were expressed as mg of quercetin equivalents (QE) per 100 g of sample using a standard curve of quercetin (0-200 mg 100 mL^{-1}). Determinations were carried out in triplicate.

8. Antioxidant activity

Extraction was accomplished as previously described for TPC and TFC analysis using ethanol:water (96:4, v/v). The antioxidant activity using the ABTS method was determined using a mother solution of $ABTS^{\bullet+}$ radical (2,2′-azinobis (3-ethylbenzothiazoline-6-sulfonic acid) in methanol (7 mM) and another one was determined to contain potassium persulfate (2.45 mM) in pH 7.0 phosphate buffer solution. The mixture was reacted at a 1:1 ratio at 25 °C in the absence of light for 16 h; subsequently, a 1950 µL of aliquot was removed from the $ABTS^{\bullet+}$ solution and 50 µL of the methanolic extract was added, mixed at 2000 rpm for 1 min in a vortex, and placed in darkness for 7 min at 25 °C [85]. The absorbance was measured at 734 nm.

The DPPH method was accomplished as previously described with some modifications [86]. A $DPPH^{\bullet}$ stock solution (2,2-diphenyl-1-picrylhydrazyl) was prepared in methanol (0.25 mM) and agitated in an ultrasonic bath. A working solution was prepared from the $DPPH^{\bullet}$ stock solution at 0.25 mM. From this solution, 1950 µL were collected to which 50 µL of the methanolic extract was added. The resulting 2000 µL solution was vortexed at 2000 rpm for 30 s and allowed to react in the dark at room temperature for 30 min. Then, the absorbance of the solution was read at 517 nm. The values of ABTS and DPPH were expressed as mg of Trolox Equivalent by 100 g^{-1} dry weight (dw).

9. Characterization of phenolic compounds

Two LC devices were used for the qualitative and quantitative analysis of phenolic compounds. The phenolic compound profiles quantified through the HPLC-DAD

system are detailed in Table 2, while the results obtained by the LC-MS system are detailed in Supplementary Table S1.

9.1. Characterization of phenolic compounds by HPLC-DAD

Chromatograms were screened at 254, 280, and 320 nm, revealing robust molar extinction coefficients. The identification process was based on comparing the retention times (Rt) and absorption spectra of all peaks across the chromatograms with those of pure standards. The HPLC-DAD parameters used for the analysis of the phenolic-rich extracts of *S. irio* samples, including linearity range, regression equations, limits of detection (LOD), limits of quantification (LOQ), and compound recoveries, are provided in Supplementary Table S3. Precision and injection repeatability tests demonstrated commendable precision in both peak areas (standard deviation <1%) and peak Rt (±2%).

HPLC analysis of phenolic compounds was carried out in a Finnigan Surveyor chromatograph equipped with a diode-array detector (DAD) and a reverse-phase C18 column (Luna® Omega, 250 mm x 4.6 mm i.d., 3 µm particle size) (Phenomenex, USA). The compounds were separated with a gradient elution using acidified water (1% acetic acid) (A) and acetonitrile (B) as mobile phase at 25 ºC. HPLC grade solvents, Merck, and Ultrapure water Milli-Q® were used for HPLC. Solvents were filtered through a 0.45 µm membrane filter (Millipore, Durapore®, Ireland) before use. The gradient started at 90% solvent A (10 min) which was reduced to 85% (20 min), 80% (10 min), 75% (10 min), 65% (10 min), 60% (10 min), 50% (20 min), and 90% (15 min). The total running time was 105 min. The flow rate was kept at 0.3 mL min^{-1} and the injection volume was 10 µL. Peaks were monitored at 254, 280, and 320 nm, and identified by retention times in comparison with pure standards: Gallic acid (97.5%, 91215), protocatechuic acid (≥97%, 37580), 4-hydroxybenzoic acid (99%, H20059), DL-*p*-hydroxyphenyllactic acid (≥97%, H3253), 3,4-dihydroxyhydrocinnamic acid (98%, 102601), chlorogenic acid (≥95%, C3878), caffeic acid (≥98%, C0625), vanillic acid (≥97%, 94770), vanillin (99%, V1104), syringic acid (≥95%, S6881), sinapic acid (≥98%, D7927), salicylic acid (≥99%, 247588), *trans*-*p*-coumaric acid (≥98%, 55823), salicylic acid (≥99%, 247588), *trans*-ferulic acid (99%, 128708), naringenin (≥95%, N5893), rutin (≥94%, R5143), rosmarinic acid (≥98%, R4033), 2-hydroxy-4-methoxybenzoic acid (99%, 173479), quercetin (≥95%, Q4951), luteolin (≥95%, GP5376), kaempferol (≥90%, K0133), apigenin (≥97%, PC15106), lithospermic acid

(≥98%, BP0878), quercetin-3-*O*-glucoside (≥95%, GC9169), and apigenin-7-*O*-glucoside (≥99%, T4S0295).

9.2. Characterization of phenolic compounds by LC-MS

Chromatographic separation was performed on a Thermo Fisher Scientific Transcend 600 LC (Thermo Scientific Transcend™, Thermo Fisher Scientific, San Jose, CA, USA) using a Hypersil Gold column (250 × 4.6 mm, 5 µm). A flow rate of 0.65 mL min^{-1} was set. The compounds were separated with gradient elution using aqueous acetic acid (acetic acid: H_2O, 1:99, *v*/*v*) (A) and methanol (B) as eluents at ambient temperature. The step gradient was as follows: 0-20 min 80% of A; then, it was linearly decreased to 25% in 10 min and remained constant during 10 min. Later, it was increased to 80% in 10 min and remained constant during 5 min. The total running time was 55 min. The column temperature was 25 °C, and the injection was 10 µL.

The LC system is coupled to a single MS Orbitrap Thermo Fisher Scientific (Exactive™, Thermo Fisher Scientific, Bremen, Germany) using an electrospray interface (ESI) (HESI-II, Thermo Fisher Scientific, San Jose, CA, USA) in positive and negative ion mode. ESI parameters were as follows: spray voltage, 4 kV; sheath gas (N_2>95%), 35 (adimensional); auxiliary gas (N_2>95%), 10 (adimensional); skimmer voltage, 18 V; capillary voltage, 35 V; tube lens voltage, 95 V; heater temperature, 305 °C; and capillary temperature, 300 °C. The mass spectra were acquired employing alternating acquisition functions: (1) full MS, ESI^+, without fragmentation (higher collisional dissociation (HCD) collision cell was switched off), mass resolving power = 25,000 FWHM; scan time = 0.25 s; (2) all-ion fragmentation (AIF), ESI^+, with fragmentation (HCD on, collision energy 30 eV), mass resolving power = 10,000 FWHM; scan time = 0.10 s; (3) full MS, ESI using the aforementioned settings; and (4) AIF, ESI using the settings explained for (2). The mass range in the full scan experiments was set at *m*/*z* 50-1000. LC chromatograms were acquired using the external calibration mode, and they were processed using Xcalibur™ version 3.0, with Qualbrowser and Trace Finder 4.0 (Thermo Fisher Scientific, Les Ulis, France). An unknown analysis was carried out with Compound Discoverer™ version 2.1.

Sensitivity and Specificity of the LC-MS Technique:

In LC-Orbitrap MS, sensitivity is often excellent due to the high-resolution capabilities of the Orbitrap mass analyzer and the sensitivity of modern LC systems. The instrument was able to detect analytes at parts-per-trillion (ppt) range.

Specificity refers to the ability of the LC-Orbitrap MS system to differentiate between analytes of interest and other compounds present in the sample matrix. The high resolution and mass accuracy of the Orbitrap mass analyzer contribute to excellent specificity by enabling precise determination of the mass-to-charge ratios (*m*/*z*) of analytes (up to 5 decimal points). Additionally, LC separation before MS analysis helps to resolve complex mixtures, further enhancing specificity.

10. Anticancer activity

The anticancer activity was determined for *S. irio* extracts. The HT-29 human colon cancer cell line and the CCD-18 colonic human myofibroblast cell lines were used to check antiproliferative activities. Cultures were supplied by the Technical Instrumentation Service of the University of Granada (Granada, Spain). First, it was checked for the absence of *Mycoplasma* and bacteria. Then, cells were grown at 37 °C and 5% CO_2 humidified atmosphere in medium RPMI-1640 supplemented with 5% fetal bovine serum, 2 mM L-Glutamine, 1 mM sodium pyruvate, 0.125 mg mL^{-1} amphotericin, and 100 mg mL^{-1} penicillin-streptomycin.

All cultures were plated in 25 cm^2 plastic tissue culture flasks (Sarstedt, USA). All culture media and reagents were purchased from Sigma-Aldrich (St. Louis, MO, USA). Cell culture and cell assay, that is, the 3-(4,5-Dimethyl-2-thiazolyl)-2,5-diphenyl-2H-tetrazolium bromide (MTT) test were accomplished as previously described [89].

In the MTT assay, cells were divided into 96-well microtiter plates, adjusted to 1×10^4 cells/well and cultivated in the medium at 37 °C, 5% CO_2 before adding the different extracts dissolved in the culture medium.

Plant extracts were prepared from fresh leaves as described in section 5 of this document. The phenolics-containing extracts were concentrated using a Buchi RE rotary evaporator equipped with a Buchi 461 water bath set at 50 °C. The concentrate was placed in a freezer and freeze-dried for 72 h using an Edwards Modulyo-4K freeze dryer (Crawley, United Kingdom). The percentage yields of the extracts for all samples were in the 1.5-1.9% range, and yields lacked statistically significant differences between the different samples (p<0.05).

The phenolics-containing extracts of *S. irio* were supplied to cells dissolved in ethanol 96% and then in the culture medium at designed concentrations (0-1000 μg mL^{-1}). The final ethanol concentration in the culture medium was maintained at 1% (v/v) to avoid solvent toxicity. To ensure experimental validity, two types of control were prepared: one contained only the culture medium and another had 1% ethanol in the culture

medium. After 48 and 72 h of cell exposure, 5 mg mL^{-1} of an MTT solution was added to the culture medium to determine the viability of cells. The absorbance was recorded at 570 nm on an enzyme-linked immunosorbent assay (ELISA) plate reader (Thermo Electron Corporation, Sant Cugat del Valles, Barcelona, Spain). The produced formazan crystals were solubilized using 100 μL dimethylsulfoxide (DMSO). Cells without phenolic extracts were considered as negative controls.

Cell survival in exposed cultures relative to unexposed cultures (negative controls) was calculated, and the number of viable cells was calculated using the following equation:

Percentage of viable cells (%) = (Absorbance of treated cells/Absorbance of untreated cells) × 100%.

The concentrations causing 50% cell growth inhibition (GI_{50}) were calculated from the growth curves. Doxorubicin (98.0-102%, D1515), from Sigma-Aldrich (Madrid, Spain) was used as a positive control, while DMSO and methanol were used as the negative (vehicle) controls. Phenolic extracts and controls were evaluated in three independent assays. Values presented are mean ± standard error of the mean. The selectivity index (SI) of extracts/compounds was calculated as the GI_{50} of the extract/compound against the CCD-18 normal cell line/GI_{50} of the same extract against the HT-29 cancer cell line. An extract with an SI value greater than 2 is considered highly selectivity against cancer cells, whereas one with an SI value less than 2 demonstrates general toxicity to normal cells [80].

References

20. Ferrón-Carrillo, F.; Guil-Guerrero, J.L.; González-Fernández, M.J.; Lyashenko, S.; Battafarano, F.; Cunha-Chiamolera, T.P.L.; Urrestarazu, M. LED enhances plant performance and both carotenoids and nitrates profiles in lettuce. *Plant Food Hum. Nutr.* **2021**, 76, 210-218. https://doi.org/10.1007/s11130-021-00894-8
32. Peçanha, D.A.; Cunha-Chiamolera, T.P.L.; Chourak, Y.; Martínez-Rivera, E.Y.; Urrestarazu, M. Effect of the matric potential on growth and water, nitrate, and potassium absorption of vegetables under soilless culture. *J. Soil Sci. Plant Nutr.* **2021**, 21, 3493-3501. https://doi.org/10.1007/s42729-021-00622-w
37. Rodríguez, D.; Reca, J.; Martínez, J.; Lao, M.T.; Urrestarazu, M. Effect of controlling the leaching fraction on the fertigation and production of a tomato crop under soilless culture. *Sci. Hortic.* **2014,** 179, 153-157. https://doi.org/10.1016/j.scienta.2014.09.030
79. Lyashenko, S.; Fabrikov, D.; González-Fernández, M.J.; Gómez-Mercado, F.; Ruiz, R.L.; Fedorov, A.; Guil-Guerrero, J.L. Phenolic composition and *in vitro* antiproliferative activity of *Borago* spp. seed extracts on HT-29 cancer cells. *Food Biosci.* **2021**, 42, 101043. https://doi.org/10.1016/j.fbio.2021.101043
80. Vichitsakul, K.; Laowichuwakonnukul, K.; Soontornworajit, B.; Poomipark, N.; Itharat, A.; Rotkrua, P. Anti-proliferation and induction of mitochondria-mediated apoptosis by *Garcinia hanburyi* resin in colorectal cancer cells. *Heliyon* **2023**, 9, e16411. https://doi.org/10.1016/j.heliyon.2023.e16411

82. Volden, J.; Bengtsson, G.B.; Wicklund, T. Glucosinolates, L-ascorbic acid, total phenols, anthocyanins, antioxidant capacities and color in cauliflower (*Brassica oleracea* L. ssp. *botrytis*); effects of long-term freezer storage. *Food Chem.* **2009**, 112, 967-976. https://doi.org/10.1016/j.foodchem.2008.07.018
83. Singleton, V.L.; Orthofer, R.; Lamuela-Raventós, R.M. Analysis of total phenols and other oxidation substrates and antioxidants by means of folin-ciocalteu reagent. *Methods Enzymol.* 1999, 299, 152-178. https://doi.org/10.1016/S0076-6879(99)99017-1
84. Zou, Y.; Lu, Y.; Wei, D. Antioxidant activity of flavonoid-rich extracts of *Hypericum perforatum* L. *in Vitro*. *J. Agric. Food Chem.* **2004**, 52, 5032-5039. https://doi.org/10.1021/jf049571r
85. Re, R.; Pellegrini, N.; Proteggente, A.; Pannala, A.; Yang, M.; Rice-Evans, C. Antioxidant activity applying an improved ABTS radical cation decolorization assay. *Free Radic. Biol. Med.* **1999**, 26, 1231-1237. https://doi.org/10.1016/s0891-5849(98)00315-3
86. Skenderidis, P.; Kerasioti, E.; Karkanta, E.; Stagos, D.; Kouretas, D.; Petrotos, K.; Tsakalof, A. Assessment of the antioxidant and antimutagenic activity of extracts from goji berry of Greek cultivation. *Toxicol. Rep.* **2018**, 5, 251-257. https://doi.org/10.1016/j.toxrep.2018.02.001
89. Ramos-Bueno, R.P.; Romero-González, R.; González-Fernández, M.J.; Guil-Guerrero, J.L. Phytochemical composition and *in vitro* anti-tumour activities of selected tomato varieties. *J. Sci. Food Agric.* **2017**, 97, 488-496. https://doi.org/10.1002/jsfa.7750
