# Supplementary material for: London Rocket (Sisymbrium irio L.) as Healthy Green: Bioactive Compounds and Bioactivity of Plants Grown in Wild and Controlled Environments
Source: Molecules. 2024 Dec 25;30(1):31. doi: 10.3390/molecules30010031 (PMC11721195; doi:10.3390/molecules30010031)
Supplement: Supplementary file 1 [file molecules-30-00031-s001.zip › Supplementary Table S1 . Phenolics compounds by LC-MS.pdf]

Supplementary Materials of the article:

London Rocket (*Sisymbrium irio* L.) as Healthy Green: Bioactive Compounds and Bioactivity of Plants Grown in Wild and Controlled Environments

Supplementary Table S1. Phenolic compounds profiles detected in *S. irio* samples by the LC-MS system

Species/ codes	4-hydroxy-benzoic acid	Vanillic acid	Procyanidin B1	Chlorogenic acid	Gallocatechin (-)	Caffeic acid	Epicatechin (-)	Delphinidine	Naringenin-7-*O*-rutinoside	Pelargonidine	*Trans-p*-coumaric acid	Kaempferol-3-*O*-rutinoside	Kaempferol-3-*O*-glucoside	Quercetin-3-*O*-glucoside
**Wild plants**														
WD	X	-	-	-	-	X	X	-	X	X	X	X	X	-
WG	-	X	-	-	X	X	-	-	X	-	-	X	-	-
WM	-	X	-	-	-	X	-	-	X	-	-	X	-	-
WT	-	-	-	-	X	X	-	-	X	X	X	X	X	-
**Growth in controlled environment plants**														
CE experiment														
C1	X	-	-	-	-	X	-	-	-	X	X	X	X	X
C2	X	-	-	-	-	X	-	-	-	X	X	X	X	X
C3	X	-	-	X	-	X	-	-	-	X	X	-	X	-
C4	X	-	-	X	-	X	-	-	X	X	X	X	X	X
C5	X	-	-	-	-	-	-	-	-	X	X	X	X	X
Lighting experiment														
L1	X	-	X	-	-	X	X	X	-	X	X	X	X	X
L2	X	-	-	-	-	X	-	-	-	X	X	-	X	X
L3	X	-	-	-	-	X	-	-	X	X	X	-	X	X
L4	X	-	-	-	-	X	-	-	X	X	X	X	X	X

Supplementary Table S1. Phenolic compounds profiles detected in *S. irio* samples by the LC-MS system (continued)

Species/ codes	Apigenin-7-*O*-rutinoside	Ferulic acid	Sinapic acid	Apigenin-7-*O*-glucoside	Apigenin-6-*C*-glucoside	Apigenin	Genistin	Isorhamnetin-3-*O*-rutinoside	Isorhamnetin-3-*O*-glucoside	Phloridzin	Naringenin	Hesperetin	Luteolin
**Wild plants**													
WD	X	X	-	X	-	-	-	X	X	-	X	-	X
WG	X	X	-	X	-	X	X	-	-	-	X	-	X
WM	X	-	-	-	-	X	X	-	-	-	X	-	X
WT	X	X	X	X	X	X	-	X	X	-	-	-	-
**Growth in controlled environment plants**													
CE experiment													
C1	X	-	X	X	-	X	-	-	X	-	X	-	X
C2	X	-	X	X	-	X	-	-	X	-	X	X	X
C3	X	-	X	X	-	X	-	-	-	-	X	-	X
C4	-	-	X	X	-	X	-	X	X	X	X	-	X
C5	X	-	X	X	-	X	-	-	X	-	X	-	X
Lighting experiment													
L1	X	-	X	X	X	X	-	X	-	-	X	-	-
L2	X	-	X	X	X	X	-	X	-	-	X	-	-
L3	X	-	X	X	X	X	-	X	-	-	X	-	-
L4	X	X	X	X	X	X	-	X	-	-	X	-	-

Comentado [MOU1]: Attention AE: Title altered

Comentado [JG2R1]: The title should be: London Rocket (*Sisymbrium irio* L.) as Healthy Green: Bioactive Compounds and Bioactivity of Plants Grown in Wild and Controlled Environments
