# Supplementary material for: London Rocket (Sisymbrium irio L.) as Healthy Green: Bioactive Compounds and Bioactivity of Plants Grown in Wild and Controlled Environments
Source: Molecules. 2024 Dec 25;30(1):31. doi: 10.3390/molecules30010031 (PMC11721195; doi:10.3390/molecules30010031)
Supplement: Supplementary file 1 [file molecules-30-00031-s001.zip › Supplementary Table S2. Glucosinolates compounds identification by HPLC-MS.pdf]

Supplementary Materials of the article:

London Rocket (*Sisymbrium irio* L.) as Healthy Green: Bioactive Compounds and Bioactivity of Plants Grown in Wild and Controlled Environments

Comentado [MOU1]: Attention AE: Title altered

Comentado [JG2R1]: The title should be: London Rocket (*Sisymbrium irio* L.) as Healthy Green: Bioactive Compounds and Bioactivity of Plants Grown in Wild and Controlled Environments

Supplementary Table S2. Glucosinolates (227 nm) detected and quantified in *S. irio* samples

Extraction	Retention time, Rt (min)	Formula	Adduct	Mass *m/z*	Common name	Chemical Class-Systematic Name
96% ethanol	4.9	$C_{10}H_{19}NO_9S_2$	$[M-H]^-$	360	Glucoputranjivin (GLS)	1-Methylethyl GSL, Isopropyl GSL
	18.7	$C_{16}H_{20}N_2O_9S_2$	$[M-H]^-$	447	Glucobrassicin (GB)	3-indolylmethyl-GSL

(*m/z*): unit for parental ion
