# Supplementary material for: London Rocket (Sisymbrium irio L.) as Healthy Green: Bioactive Compounds and Bioactivity of Plants Grown in Wild and Controlled Environments
Source: Molecules. 2024 Dec 25;30(1):31. doi: 10.3390/molecules30010031 (PMC11721195; doi:10.3390/molecules30010031)
Supplement: Supplementary file 1 [file molecules-30-00031-s001.zip › Supplementary Table S3. Chromatography parameters HPLC-DAD.pdf]

Supplementary Materials of the article:

London Rocket (*Sisymbrium irio* L.) as Healthy Green: Bioactive Compounds and Bioactivity of Plants Grown in Wild and Controlled Environments

Supplementary Table S3. HPLC-DAD parameters used for the analysis of phenolic-rich extracts of *Sisymbrium irio* samples[a]

Nº	Rt (min)	Phenolic compound	Detection wavelength (nm)	Linear range (µg mL^{-1}) and correlation coefficients (R^2)	Repeatability (%RSD)	LOD (µg mL^{-1})	LOQ (µg mL^{-1})	Recovery (%)
1	13.00	Gallocatechin (−)	280	-	-	-	-	-
2	21.30	Protocatechuic acid (3,4-dihydroxybenzoic acid)	254	5-50 (0.991)	4.43	0.18	0.45	98.20
3	56.20	*Trans*-*p*-coumaric acid	320	5-50 (0.990)	6.80	0.30	0.60	97.15
4	60.40	*Trans*-ferulic acid	254	5-50 (0.991)	6.59	0.40	0.80	98.97
5	64.10	Isoquercetin (Quercetin-3-*O*-glucoside)	254	5-50 (0.995)	4.76	0.50	0.90	96.99
6	66.80	Apigetrin (Apigenin-7-*O*-glucoside)	320	10-100 (0.994)	5.60	0.30	0.90	98.34
7	71.20	Isorhoifolin (Apigenin-7-*O*-rutinoside)	280	-	-	-	-	-
8	76.10	Isorhamnetin-3-*O*-glucoside	280	-	-	-	-	-
9	78.25	Luteolin	254	10-100 (0.999)	6.97	0.30	0.50	100.20
10	80.20	Nicotiflorin (Kaempferol-3-*O*-rutinoside)	320	-	-	-	-	-
11	84.50	Naringenin	280	5-50 (0.993)	7.55	0.40	0.50	99.89
12	85.60	Apigenin	320	10-100 (0.996)	5.91	0.20	0.70	97.09

[a] Detection wavelength (nm) for compounds 1, 7, 8, and 10 were taken from the literature, and Rt was tentatively assigned to selected peaks in the HPLC-DAD chromatogram.
