# Supplementary material for: London Rocket (Sisymbrium irio L.) as Healthy Green: Bioactive Compounds and Bioactivity of Plants Grown in Wild and Controlled Environments
Source: Molecules. 2024 Dec 25;30(1):31. doi: 10.3390/molecules30010031 (PMC11721195; doi:10.3390/molecules30010031)
Supplement: Supplementary file 1 [file molecules-30-00031-s001.zip › Supplementary Table S4. Phenolic parameters LC-MS and identification basis.pdf]

Supplementary Materials of the article:

London Rocket (*Sisymbrium irio* L.) as Healthy Green: Bioactive Compounds and Bioactivity of Plants Grown in Wild and Controlled Environments

Comentado [MOU1]: Attention AE: Title altered

Comentado [JG2R1]: The title should be: London Rocket (*Sisymbrium irio* L.) as Healthy Green: Bioactive Compounds and Bioactivity of Plants Grown in Wild and Controlled Environments

Supplementary Table S4. Phenolic compounds identified by LC-MS in the ethanolic extracts of *Sisymbrium irio* leaves and identification basis

Phenolic compound	Retention time (min)	Formula	Adduct	Mass *m/z* [a]	*m/z* (Delta) (ppm)	Fragment [b]	Identification basis
4-hydroxybenzoic acid	5.40	$C_7H_6O_3$	$[M-H]^-$	137.02362	-5.83396	93.03348	Molecular ion $[M-H]^-$ *m/z* 137 and its characteristic product ion 93, generated by the loss of the CO_2 group
Vanillic acid	6.31	$C_8H_8O_4$	$[M-H]^-$	167.03498	-3.96913	152.00996	Molecular ion $[M-H]^-$ *m/z* 167 and its characteristic product ion 152 due to the loss of CH_4
Procyanidin B1	6.45	$C_{30}H_{26}O_{12}$	$[M-H]^-$	577.13495	-0.34874	289.07208	Molecular ion $[M-H]^-$ *m/z* 577 and the fragment ion at *m/z* 289, formed through quinone methide fragmentation (QM) of the parent ion.
Chlorogenic acid	6.59	$C_{16}H_{18}O_9$	$[M-H]^-$	353.08792	1.43837	191.02567	Molecular ion $[M-H]^-$ *m/z* 353 and a fragment of *m/z* 191 ($C_7H_{11}O_6$) corresponding to [Quinic acid-H]
Gallocatechin (-)	6.67	$C_{15}H_{14}O_7$	$[M+H]^+$	307.08123	-2.26035	139.03884	Molecular ion $[M+H]^-$ *m/z* 307, and fragment *m/z* 139, that is probably produced due to the cleavage of C-ring via Retro Diels-Alder (RDA) mechanism fission [91]
Caffeic acid	6.81	$C_9H_8O_4$	$[M-H]^-$	179.03448	-2.76559	135.04436	Molecular ion $[M-H]^-$ *m/z* 179 and its characteristic product ion 135, due to the loss of the CO_2 group
Epicatechin (-)	7.00	$C_{15}H_{14}O_6$	$[M+H]^+$	291.08615	-0.54581	139.03882	Molecular ion $[M+H]^-$ *m/z* 291, and fragment *m/z* 139, that is probably produced due to the cleavage of C-ring via Retro Diels-Alder (RDA) mechanism fission [91]
Delphinidine	7.47	$C_{15}H_{10}O_7$	$[M+H]^+$	303.05054	2.00333	257.04648	Molecular ion $[M-H]^+$ *m/z* 303, and fragment ion $[M-CO-H_2O]^+$ (*m/z* 257)
Narirutin (Naringenin-7-*O*-rutinoside)	7.55	$C_{27}H_{32}O_{14}$	$[M+H]^+$	581.18648	3.08104	271.06211	Molecular ion $[M+H]^+$ *m/z* 581 and *m/z* 271, due to the loss of 308 Da (rutinose)
Pelargonidine	7.56	$C_{27}H_{30}O_{15}$	$[M+H]^+$	595.16528	-2.42514	271.06010	Molecular ion $[M+H]^+$ *m/z* 595, and fragment ion at *m/z* 271, attributed to pelargonidine, that correspond to the loss of two hexoses ($[M+H-324]^+$)
Trans-p-coumaric acid	7.73	$C_9H_8O_3$	$[M-H]^-$	163.03946	-3.74614	119.04927	Molecular ion $[M-H]^-$ *m/z* 163 and its characteristic product ion 119, due to the loss of the CO_2 group
Kaempferol-3-*O*-rutinoside	8.19	$C_{27}H_{30}O_{15}$	$[M-H]^-$	593.15167	0.81322	285.04099	Molecular ion $[M-H]^-$ *m/z* 593, and *m/z* 285 corresponding to a deprotonated kaempferol aglycone, and further loss of the rutinoside

							moiety
Astragalin (kaempferol-3-*O*-glucoside)	8.21	$C_{21}H_{20}O_{11}$	$[M-H]^-$	447.09369	0.91472	255.02924	Molecular ion $[M-H]^-$ *m/z* 447 and *m/z* 255, corresponding to the loss of CH_2O from the aglycone (30 Da) [92]
Isoquercetin (Quercetin-3-*O*-glucoside)	8.23	$C_{21}H_{20}O_{12}$	$[M-H]^-$	463.08820	2.52640	302.03699	Retention time after the "−7−*O*− isomer", molecular ion $[M-H]^-$ *m/z* 463, and *m/z* 302, corresponding to the aglycone of quercetin following the loss of a hexose ($[M-H-162]^-$
Isorhoifolin (Apigenin-7-*O*-rutinoside)	8.45	$C_{27}H_{30}O_{14}$	$[M+H]^+$	579.17083	-2.30566	271.05913	Molecular ion $[M+H]^+$ *m/z* 579, and *m/z* 271, due to the loss of 308 Da (rutinose)
Trans-ferulic acid	8.50	$C_{10}H_{10}O_4$	$[M-H]^-$	193.05063	−2.45718	134.03690	Molecular ion $[M-H]^-$ *m/z* 193, and *m/z* 134 corresponding to the loss of CO_2 and CH_3
Sinapic acid	8.51	$C_{11}H_{12}O_5$	$[M-H]^-$	223.06120	-1.28626	121.02821	Molecular ion $[M-H]^-$ m/z 223, and the loss of $2CH_3$-CO_2-CO (*m/z* 121) [93]
Apigenin-7-*O*-glucoside	8.57	$C_{21}H_{20}O_{10}$	$[M+H]^+$	433.11292	-2.54805	271.05963	Molecular ion $[M+H]^-$ *m/z* 433, and *m/z* 271 corresponding to the aglycon apigenin, by the loss of glucose (162 Da)
Isovitexin (Apigenin-6-*C*-glucoside)	8.58	$C_{21}H_{20}O_{10}$	$[M-H]^-$	431.09837	0.39726	311.05586	Molecular ion $[M-H]^-$ *m/z* 431, and *m/z* $311[M-H-120]^-$ that correspond to the loss of sugar, typical of C-linked hexoside group
Apigenin	8.59	$C_{15}H_{10}O_5$	$[M+H]^+$	271.06010	-2.96741	153.01779	Molecular ion $[M+H]^-$ *m/z* 271, and *m/z* 153, which refers to $^{1,3}A^+$, which is formed as a result of the cleavage of the bond between the 1st and 3rd carbon atoms of the middle ring (C-ring), while the charge is retained on the A-ring side of the compound [94]
Genistin	8.60	$C_{21}H_{20}O_{10}$	$[M+H]^+$	433.11292	-0.57514	271.05276	Molecular ion $[M-H]^+$ at *m/z* 433 and fragment *m/z* 271 $[M-H-162]^+$ via the loss of a glucose moiety [95]
Narcissin (isorhamnetin-3-*O*-rutinoside	8.66	$C_{28}H_{32}O_{16}$	$[M-H]^-$	623.16199	0.36476	315.05139	Molecular ion $[M-H]^-$ at *m/z* 623 and fragment *m/z* 315 $[M-H-308]^-$ due to the loss of rutinose
Isorhamnetin-3-*O*-glucoside	8.67	$C_{22}H_{22}O_{12}$	$[M+H]^+$	479.11774	-5.33328	314.04263	Molecular ion $[M+H]^+$ *m/z* 479, and fragment *m/z* 314, due to the loss of a hexose (162 Da)
Phloridzin (phloretin-2-glucoside)	8.83	$C_{21}H_{24}O_{10}$	$[M-H]^-$	435.13113	3.36459	167.03427	Molecular ion $[M-H]^-$ *m/z* 435, and fragment *m/z* 167 corresponding to $[M-H-C_8H_7O_4]^-$ [96]
Naringenin	9.19	$C_{15}H_{12}O_5$	$[M-H]^-$	271.06158	1.41840	119.04926	Molecular ion $[M-H]^-$ *m/z* 271, and *m/z* 119 that correspond to fragmentation of the B ring ($^{1,3}B^-$), as described by Fabre et al. (2001) [97]
Hesperetin	9.21	$C_{16}H_{14}O_6$	$[M-H]^-$	301.07196	0.66579	164.01068	Molecular ion $[M-H]^-$ at *m/z* 301 and fragment *m/z* 164, that corresponds to $[M-H-C_7H_5O_3]^-$ [98]
Luteolin	9.24	$C_{15}H_{10}O_6$	$[M-H]^-$	285.04083	1.30690	133.02834	Molecular ion $[M-H]^-$ *m/z* 285, and *m/z* 175, corresponding to the loss of C_3O_2 - C_2H_2O [99]

[a] Mass error lower than 5 ppm; [b] Mass error lower than 10 ppm.

References

91. Rawat, P.; Singh, Y.; Tiwari, S.; Mishra, D.K.; Kanojiya, S. The characterization and quantification of structures of *Cajanus scarabaeoides* phytochemicals and their seasonal variation analysis using ultra-performance liquid chromatography-tandem mass spectrometry. *Rapid Commun. Mass Spectrom.* **2023**, 37, e9440. https://doi.org/10.1002/rcm.9440
92. Dantas, C.A.G.; Abreu, L.S.; da Cunha, H.N.; Veloso, C.A.G.; Souto, A.L.; Agra, M.F.; Costa, V.C.O.; da Silva, M.S.; Tavares, J.F. Dereplication of phenolic derivatives of three Erythroxylum species using liquid chromatography coupled with ESI-MS^n and HRESIMS. *Phytochem. Anal.* **2021**, 32, 1011-1026. https://doi.org/10.1002/pca.3043
93. Marcum, C.L.; Jarrell, T.M.; Zhu, H.; Owen, B.C.; Haupert, L.J.; Easton, M.; Hosseinaei, O.; Bozell, J.; Nash, J.J.; Kenttämaa, H.I. A fundamental tandem mass spectrometry study of the collision activated dissociation of small, deprotonated molecules relaed to lignin. *ChemSusChem* **2016**, 9, 3513-3526. https://doi.org/10.1002/cssc.201600678.
94. Abrankó, L.; Szilvássy, B. Mass spectrometric profiling of flavonoid glycoconjugates possessing isomeric aglycones. *J. Mass Spectrom.* **2015**, 50, 71-80. https://doi.org/10.1002/jms.3474
95. Liang, Y.; Zhao, W.; Wang, C.; Wang, Z.; Wang, Z.; Zhang, J. A comprehensive screening and identification of genistin metabolites in rats based on multiple metabolite templates combined with UHPLC-HRMS analysis. *Molecules* **2018**, 23, 1862. https://doi.org/10.3390/molecules23081862
96. Wu, F.P.; Liu, L.H.; Jin, P.; Pu, H.; Cai, W. Determination of metabolites of phloretin in rats using UHPLC-LTQ-Orbitrap mass spectrometry. *Trop. J. Pharm. Res.* **2019**, 18, 2167-2173. https://doi.org/10.4314/tjpr.v18i10.24
97. Fabre, N.; Rustan, I.; de Hoffmann, E.; Quetin-Leclercq, J. Determination of flavone, flavonol, and flavanone aglycones by negative ion liquid chromatography electrospray ion trap mass spectrometry. *J. Am. Soc. Mass Spectrom.* **2001**, 12, 707-715. https://doi.org/10.1016/S1044-0305(01)00226-4.
98. Yehia, S.M.; Ayoub, I.M.; Watanabe, M.; Devkota, H.P.; Singab, A.N.B. Metabolic profiling, antioxidant, and enzyme inhibition potential of *Iris pseudacorus* L. from Egypt and Japan: A comparative study. *Sci. Rep.* **2023**, 13, 5233. https://doi.org/10.1038/s41598-023-32224-0
99. Śliwka-Kaszyńska, M.; Anusiewicz, I.; Skurski, P. The mechanism of a retro-diels–alder fragmentation of luteolin: theoretical studies supported by electrospray ionization tandem mass spectrometry results". *Molecules* **2022**, 27, 1032. https://doi.org/10.3390/molecules27031032
