# Supplementary material for: London Rocket (Sisymbrium irio L.) as Healthy Green: Bioactive Compounds and Bioactivity of Plants Grown in Wild and Controlled Environments
Source: Molecules. 2024 Dec 25;30(1):31. doi: 10.3390/molecules30010031 (PMC11721195; doi:10.3390/molecules30010031)
Supplement: Supplementary file 1 [file molecules-30-00031-s001.zip › Supplementary Table S5. Nutrient solution.pdf]

Supplementary Materials of the article:

London Rocket (*Sisymbrium irio* L.) as Healthy Green: Bioactive Compounds and Bioactivity of Plants Grown in Wild and Controlled Environments

Comentado [MOU1]: Attention AE: Title altered

Comentado [JG2R1]: The title should be: London Rocket (*Sisymbrium irio* L.) as Healthy Green: Bioactive Compounds and Bioactivity of Plants Grown in Wild and Controlled Environments

Supplementary Table S5. Composition of the nutrient solutions used for culturing *Sisymbrium irio* plants[a]

Electrical conductivity ($dS\ m^{-1}$)	pH	Macronutrients (mM)						Micronutrients (µM)					
		NO_3^-	$H_2PO_4^-$	SO_4^{2-}	K^+	Ca^{2+}	Mg^{2+}	Fe	Mn	Cu	Zn	B	Mo
2.0	5.8	10.25	1.50	1.75	4.75	5.00	1.51	15	10	0.75	5	30	0.5
2.5	5.8	12.81	1.88	2.19	5.95	6.25	1.89	15	10	0.75	5	30	0.5
3.0	5.8	15.37	2.26	2.63	7.14	7.50	2.27	15	10	0.75	5	30	0.5
3.5	5.8	17.93	2.64	3.07	8.33	8.75	2.65	15	10	0.75	5	30	0.5
4.0	5.8	20.49	3.02	3.51	9.52	10.0	3.03	15	10	0.75	5	30	0.5

[a] Based on Sonneveld and Straver (1994) [90].
