# Supplementary material for: London Rocket (Sisymbrium irio L.) as Healthy Green: Bioactive Compounds and Bioactivity of Plants Grown in Wild and Controlled Environments
Source: Molecules. 2024 Dec 25;30(1):31. doi: 10.3390/molecules30010031 (PMC11721195; doi:10.3390/molecules30010031)
Supplement: Supplementary file 1 [file molecules-30-00031-s001.zip › Supplementary Table S6. Parameters of LED.pdf]

Supplementary Materials of the article:

London Rocket (Sisymbrium irio L.) as Healthy Green: Bioactive Compounds and Bioactivity of Plants Grown in Wild and Controlled Environments

Supplementary Table S6. Parameters of sun and LED spectra used during rooting phase and growing phase of *Sisymbrium irio* L. plants

Parameters	Solar spectrum	L1<br>L18 T8 Roblan®	L2<br>L18 AP67 Valoya®	L3<br>L18 NS1 Valoya®	L4<br>L18 NS12 Valoya®
Illuminance (lux)	40,000 ± 1,500[a]	4086 ± 374[c]	2231 ± 106[d]	4685 ± 291[b]	4778 ± 297[b]
Photosynthetic photon flux (μmol $m^{-2}s^{-1}$)	400 ± 40[a]	95.0 ± 10.5[c]	66.0 ± 3.8[d]	109.9 ± 5.9[b]	112.0 ± 6.0[b]
Spectral fraction (%)					
UV	4.0 ± 0.3[a]	0.13 ± 0.02[c]	0.09 ± 0.01[c]	0.26 ± 0.01[b]	0.26 ± 0.01[b]
Blue	20.2 ± 0.4[b]	26.3 ± 1.3[a]	12.2 ± 0.1[c]	20.5 ± 0.6[b]	20.9 ± 0.7[b]
Green	31.8 ± 0.8[c]	42.9 ± 0.5[a]	19.3 ± 0.5[d]	37.3 ± 0.4[b]	37.3 ± 0.4[b]
Red	30.7 ± 0.5[c]	26.9 ± 1.6[c]	53.3 ± 0.3[a]	35.4 ± 0.7[b]	35.7 ± 0.7[b]
FR	13.3 ± 0.4[b]	3.6 ± 0.2[d]	15.2 ± 0.3[a]	6.5 ± 0.2[c]	6.6 ± 0.2[c]

PPF = Photosynthetic photon flux. Different letters within a row indicate significant differences ($p < 0.05$)
